# Green nanopriming: responses of alfalfa (*Medicago sativa* L.) seedlings to alfalfa extracts capped and light-induced silver nanoparticles

**DOI:** 10.1186/s12870-022-03692-9

**Published:** 2022-07-05

**Authors:** Kexiao Song, Donghao Zhao, Haoyang Sun, Jinzhu Gao, Shuo Li, Tianming Hu, Xueqing He

**Affiliations:** College of Grassland Agriculture, Northwest A&F University, Yangling, 712100 Shaanxi Province China

**Keywords:** Green nanopriming, Light-induced AgNPs, Alfalfa growth, Antioxidant enzyme system

## Abstract

The application of nanotechnology in agriculture can remarkably improve the cultivation and growth of crop plants. Many studies showed that nanoparticles (NPs) made plants grow more vigorously. Light can make NPs aggregated, leading to the reduction of the NPs toxicity. In addition, treatment with NPs had a “hormesis effect” on plants. In this study, light-induced silver nanoparticles (AgNPs) were synthesized by using the alfalfa (*Medicago sativa* L.) extracts, and then the optimal synthetic condition was determined. Light-induced AgNPs were aggregated, spherical and pink, and they were coated with esters, phenols, acids, terpenes, amino acids and sugars, which were the compositions of alfalfa extracts. The concentration of free Ag^+^ was less than 2 % of the AgNPs concentration. Through nanopriming, Ag^+^ got into the seedlings and caused the impact of AgNPs on alfalfa. Compared with the control group, low concentration of light-induced AgNPs had a positive effect on the photosynthesis. It was also harmless to the leaf cells, and there was no elongation effect on shoots. Although high concentration of AgNPs was especially beneficial to root elongation, it had a slight toxic effect on seedlings due to the accumulation of silver. With the increase of AgNPs concentration, the content of silver in the seedlings increased and the silver enriched in plants was at the mg/kg level. Just as available research reported the toxicity of NPs can be reduced by using suitable synthesis and application methods, the present light induction, active material encapsulation and nanopriming minimized the toxicity of AgNPs to plants, enhancing the antioxidant enzyme system.

**Graphical Abstract:**

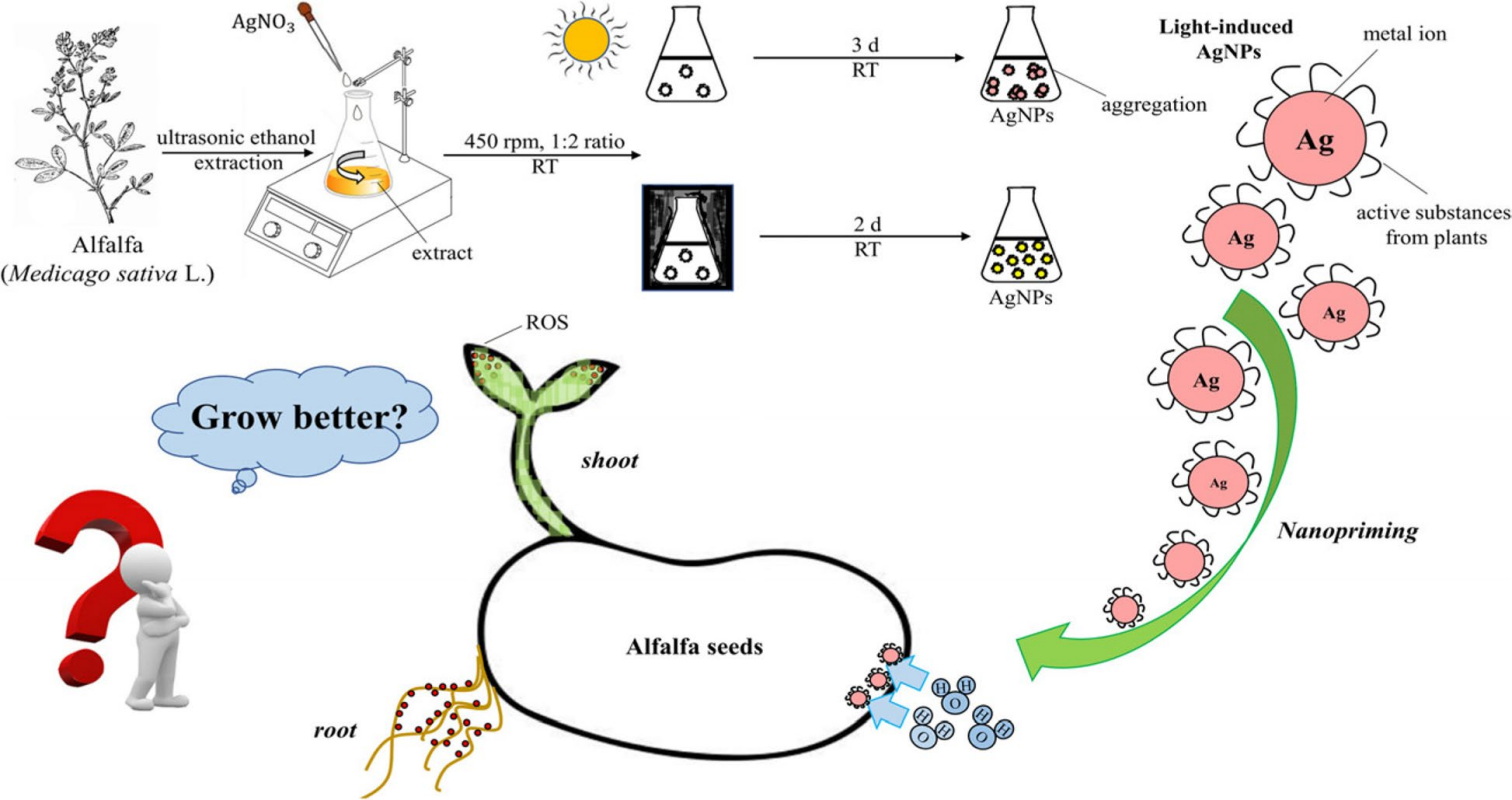

**Supplementary Information:**

The online version contains supplementary material available at 10.1186/s12870-022-03692-9.

## Background

Nanoparticles (NPs) are solid particles of atoms or molecules with size between 1-100 nm. Owing to their size and shape, NPs have some excellent physical properties compared with macromolecules [1]. Among all types of NPs, metal NPs are widely used in various scientific and technological researches due to their excellent properties, such as a large surface area to volume ratio and high dispersibility in solution [2, 3]. In addition, nanotechnology is one of the advanced techniques in agricultural applications, including nano-fertilizers, intelligent drug delivery and nano-pesticides [4]. In general, conventional NPs can be made by physical, chemical and green-synthesized methods [5]. Physical and chemical methods require energy-intensive, multi-step processes or toxic chemicals, so they have many limitations [6–8]. The green-synthesized method has attracted more and more attention because of its low cost and environmental friendliness [9]. As for green synthesis, a general method widely adopted is to use plant extracts to synthesize NPs, that its characteristics is low-cost, environmentally friendly and biocompatible [10, 11]. In this method, several parts of plants (such as leaves, stems, roots, etc.) are washed with distilled water, and then boiled in general solvent (distilled water) to obtain the plant extracts. The extracts are simply mixed with the metallic salt solution at a specific temperature, and then metal ions are converted into NPs, all within a few minutes in an eco-friendly way [12].

Recently, various plants have been successfully used to synthesize many kinds of AgNPs, such as the leaves of *Erythrina suberosa* L. [13], the roots of *Phoenix dactylifera* L. [14], the flowers of *Malva sylvestris* L. [15] and *Phyllanthus emblica* L. [16]. These studies demonstrated that the extracts of plants can be used to synthesize AgNPs [17]. It was because that plant phytochemicals had great reduction and stabilization [18], such as polyphenols, flavonoids, organic acids, alkaloids and other antioxidant components [19]. Therefore, using alfalfa extracts to obtain AgNPs is feasible in principle. As for biological effects, its different forms determined special physical and chemical properties [20]. In addition, silver is not an essential nutrient element for plants, and its toxicity to plants is known to all under high concentration. Therefore, we tried to explore the reduction of toxicity by this synthesis, which could also illustrate how metal NPs affected plants.

Alfalfa (*Medicago sativa* L.) is a major perennial legume forage crop that is widely grown worldwide due to its economic importance and outstanding agricultural traits [21]. However, there are few studies on the application of metal NPs to forages. We can make a comprehensive assessment with the effect of other plants on AgNPs. Applying AgNPs to plants has both positive and negative effects, depending on different concentrations of AgNPs. From a biological point of view, particularly high concentrations of AgNPs would not be used to treat plants [22, 23]. As for positive effects, previous reports had demonstrated that proper concentration of AgNPs can be used to enhance corn (*Zea mays* L.) [24] and rice (*Oryza sativa* L.) [25] seed germination rate, as well as onion (*Allium cepa* L.) seedlings growth and biomass [26], and low concentration of AgNPs promoted root elongation, shoot length, root length and chlorophyll content of *Hordeum vulgare* L. [22]. As for negative effects, some studies had showed that high concentration of AgNPs can affect the photosynthesis [27] and reduce the total chlorophyll content and increase the oxidative stress parameters significantly [28]. The application of AgNPs on plants seems to be a double-edged sword. Besides concentration, phytotoxicity of AgNPs also depends on the application method, exposure time, size, chemical composition of the NPs, capping agent and presence or absence of aggregation [29]. Not only that, it is necessary to ensure that NPs have low toxicity before they are placed in the environment. Fratoddi [30] thought the NPs synthesized using chemicals had significant toxicity in vivo and in vitro, and appropriate coating could reduce its toxicity. Moreover, another study also discovered that NPs synthesized by plants were less toxic than those in chemical methods because of a layer of phytochemicals [9]. In addition, the metal core of NPs had a certain degree of toxicity, but if the ion content was low enough, which could arouse the stress resistance of plants [24, 26].

Currently, nanopriming is a new type of seed priming technology that has many great advantages: having low exposure time, breaking seed dormancy, improving seed vigor and maximizing germination potential [31, 32]. Although silver is not an essential nutrient for plants, the green synthetic AgNPs periphery covered with active substances provides exactly a small amount of nutrients to plants. Moreover, different from continuous fertilization of AgNPs, a short time soaking can reduce the accumulation of NPs in plants [33]. In view of the positive effects of green-synthesized AgNPs on plants, we used green-synthesized technology to obtain nanoparticles and applied them to alfalfa seeds. We call this technology—green nanopriming. The objectives of our study were: (1) Obtain a green and low-energy consumption method to synthesize AgNPs with alfalfa extracts; (2) Discuss the feasibility of this synthesis method to reduce the toxicity of NPs; (3) Apply the synthesized AgNPs to seeds by nanopriming, observing the effects on alfalfa and providing a theoretical basis for the application of NPs of other metallic elements.

## Materials and methods

### Green synthesis of AgNPs from alfalfa extracts

The aerial parts of “SARDI 10” alfalfa at latter stage of blooming were harvested in the experimental field (34°17′38.95″N, 108°04′31.49″E), Northwest A&F University, Yangling, Shaanxi province, China. The collected parts were first dried at 378.15 K for 30 minutes, followed by 338.15 K for 2 days. Then, they were pulverized to get grass powder whose size was less than 60 mesh. The obtained grass powder was sealed at room temperature. For the extract process, first, 1 g of grass powder was soaked with 60% ethanol at a ratio of 1: 30 kg/L for 1 hour. Then, the reaction was proceeded by sonication with a power of 4×10^4^ Hz for 30 minutes at 323.15 K. Finally, the mixture was filtered at room temperature and diluted to a volume of 50 mL with 60% ethanol. The alfalfa extracts were stored at 277.15 K.

To perform the green synthesis of AgNPs, first, 2×10^-3^ mol/L AgNO_3_ and alfalfa extracts were mixed and stirred at room temperature for 30 minutes, protected from light. The volume ratio (AgNO_3_: alfalfa extracts) was chosen to be 1:4, 1:2, 1:1, 2:1 and 4:1, and the rotation speed was set to 150, 250, 350 and 450 rpm. Next, the reaction solutions were divided into two equal portions, and they were placed in the dark and in the sunlight for several days, respectively. Then, the solutions were centrifuged to obtain green-synthesized AgNPs. After that, they were washed three times with deionized water. Finally, AgNPs were dried in drying dish under vacuum for 24 hours and stored at room temperature in the dark.

### Characterization of green-synthesized AgNPs

The reduction of Ag^+^ to the nanoparticle was monitored by measuring the UV-visible spectrum of the solutions. The UV-visible spectrum was recorded on spectrophotometer (Shimadzu UV-3900 UV-VIS Spectrophotometer, Tokyo, Japan). All the measurements were performed within the range of 300-800 nm at a resolution of 1 nm. The absorption peak around 400 nm was used to identify the generated AgNPs. To determining the shape and size of AgNPs, the AgNPs were dissolved in ethanol, and sonicated for about 15 minutes. A drop of the filtered sample was placed on a Cu grid and dried under vacuum for analysis. The prepared samples were performed on Transmission electron microscope (TEM) (JEM-1230, Japan) at an accelerating voltage of 120 kV. Crystalline nature of green-synthesized AgNPs was determined by X-ray diffraction (XRD) analysis. First, the powder of AgNPs was passed through 200 mesh. Then it was subjected to X-ray diffractometer (Bruker, Model D8 ADVANCE A25, Germany) at 40 kV and 40 mA with Cu Kα1 radiation. The scan 2θ range was 10-80°. After the AgNPs were dissolved in absolute ethanol, alfalfa extracts and AgNPs were tested by ATR for Fourier transform infrared (FTIR) spectroscopy (Thermo Scientific, Model Nicolet iS10, USA) to detect the functional groups involved in synthesis of AgNPs. The spectrum was recorded with a resolution of 0.4 cm^-1^ and a scanning range of 450 to 4000 cm^-1^.

### Quadrupole-Orbitrap high-resolution LC-MS pair

In order to analyze chemical compounds in green-synthesized AgNPs, a non-targeted metabolomics approach was developed by using liquid chromatograph-mass spectrometer (LC-MS). The sample was filtered with 0.22 μm filter membrane and analysed on the LC (Thermo Scientific, Model UltiMate 3000 RS, USA) and MS (Thermo Scientific, Model Q Exactive, USA). In brief, the separation of chemical components was achieved on the XB-C18 (50×2.1 mm, 1.8 μm, Welch) column with a flow rate of 0.3 mL/min. The used gradient mobile phases were (A) water phase: 0.1 % formic acid aqueous solution, and (B) organic phase: methanol. The following gradient system was applied for the elution compounds: 0 min, 2% B; 5 min, 20% B; 10 min, 50% B; 15 min, 80% B; 20 min, 95% B; and 26 min, 2% B. The data was collected by the high-resolution liquid quality, and then the database was searched and compared. MS detection was achieved at ESI, and model was full mass/dd-MS2. The spray voltage was 3.8 kV (positive), and the temperature was set to 300 °C. The pressure of the nitrogen nebulizing gas was set to 310 kPa. Data was recorded in centroid mode in the range from m/z 150-2000.

### Ag^+^ releases from AgNPs by ICP-OES

The concentration of Ag^+^ was measured by inductively coupled plasma optical emission spectrometer (ICP-OES; Agilent, 725-ES, Australia). Stock suspensions of different AgNPs were put into each filter tube. Tubes were then centrifuged for 30 minutes, 2500 rpm, and the filtrate was digested in 5% HNO_3_ for at around 24 hours and analyzed by ICAP7000.

### Plant culture and treatment

The mature “Ju Neng 7” seeds of alfalfa were purchased from Beijing Clover Group in February, 2021. Randomly selected seeds were disinfected with 75 % alcohol for 30 s and washed 5 times with sterile water. There were 6 treatments with light-induced AgNPs (12.5, 25, 50, 100, 200 mg/L) and H_2_O. Moreover, there were three samples in each treatment, and there were 50 seeds in each sample. The treated seeds were soaked in the corresponding solution for 3 hours, and then dried at room temperature for 48 hours. For each treatment, the seeds were germinated in Petri dishes on 2-layer filter paper imbibed with 5 mL sterile water. Then the seeds were put in a germination chamber at 298.15 ± 275.15 K, 85 % RH and a 16/8-hour photoperiod (light/dark) with 10000 Lx irradiance for 14 days. A certain amount of water was replenished every day in order to make seeds grow.

### The effect of green-synthesized AgNPs on seeds

On the 4th and 14th days, germination percentages were calculated. Water uptake by the seeds after imbibition were calculated for 2, 4, 6, 8, 10, 24 and 36 hours according to the method of Mahakham [25]. The α-amylase enzyme activity of the germinating seeds was determined according to the approach of Hashemi [34]. A unit of α-amylase enzyme activity was defined as catalyzing the production of 1 mg reducing sugar per gram of tissue per minute. The whole seed and longitudinally cut cross-section were sprayed with gold to observe the seed epidermis treated with AgNPs under scanning electron microscope (SEM) (FEI, Model Nano SEM-450, USA). Energy dispersive spectrometer (EDS) analysis was performed on the longitudinally cut seeds.

### Seedling growth assay

After 14 days of seed germination, the shoot length, root length, fresh weight (FW) and dry weight (DW) of all seedlings were measured. Fresh leaves were soaked in 100 % ethanol for 24 hours to extract chlorophyll, and then they were ground for 60 s. The chlorophyll content was determined at the wavelengths of 665 nm and 649 nm. The values of chlorophyll content were expressed as mg/g of fresh weight [35, 36]. Following the method of Kannaujia [37], we calculated the seedling vigor index (SVI), germination index (GI) and relative root elongation (RRE):

$$\mathrm{Relative}\kern0.17em \mathrm{root}\kern0.17em \mathrm{elonggation}\;\left(\mathrm{RRE}\right)=\frac{\left(\mathrm{Mean}\kern0.17em \mathrm{root}\kern0.17em \mathrm{length}\kern0.17em \mathrm{with}\kern0.17em \mathrm{AgNPs}\right)}{\mathrm{Mean}\kern0.17em \mathrm{root}\kern0.17em \mathrm{length}\kern0.17em \mathrm{of}\kern0.17em \mathrm{control}}\times 100\%$$

$$\mathrm{RGermination}\ \mathrm{index}\ \left(\mathrm{GI}\right)=\frac{\left(\mathrm{Relative}\ \mathrm{seed}\ \mathrm{germination}\right)\times \left(\mathrm{Relative}\ \mathrm{root}\ \mathrm{elongation}\right)}{100}$$

$$\mathrm{Seedling}\ \mathrm{vigor}\ \mathrm{index}\ \left(\mathrm{SVI}\ \mathrm{I}\right)=\mathrm{Germination}\ \mathrm{percantage}\times \left(\mathrm{Root}+\mathrm{Shoot}\ \mathrm{length}\right)$$

$$\mathrm{Seedling}\ \mathrm{vigor}\ \mathrm{index}\ \left(\mathrm{SVI}\ \mathrm{II}\right)=\mathrm{Germination}\ \mathrm{percantage}\times \left(\mathrm{Root}+\mathrm{Shoot}\ \mathrm{DW}\right)$$

### Root activity and leaf cell viability determination

Root activity was determined by triphenyl tetrazolium chloride (TTC) method [38]. Membrane integrity of 14 d alfalfa leaves was assessed by Evans Blue assay, according to the method of Baker and Mock [39]. Finally, the absorbance of the solution was measured at 600 nm to detect whether the cells in leaves were dead.

### Determination of antioxidant enzyme system in shoots and roots

The influence of AgNPs on antioxidative enzyme activity was studied from the alfalfa seedlings after 14 days growth. Superoxide dismutase (SOD, EC 1.15.1.1) activity was calculated as unit enzyme required for 50 % inhibition of NBT per gram fresh tissue per minute according to the approach of Beauchamp and Fridovich [40]. Catalase (CAT, EC 1.11.1.6) activity was measured according to the approach of Aebi [41]. The reduction in absorbance due to H_2_O_2_ degradation was measured at 240 nm. The amount of 0.1 decrease in A_240_ within 1 minute was a unit of enzyme activity. Peroxidase (POD, EC 1.11.1.7) activity was analyzed following the method of Hemeda and Klein [42]. An increase of 0.01 in A_470_ per minute was a unit of enzyme activity.

### Determination of proline and malondialdehyde (MDA) in shoots and roots

Proline content was determined after 14 days growth of alfalfa seedlings according to the method of Bates [43]. The absorbance of chromophore containing toluene was recorded by spectrophotometer at 520 nm. Proline content was measured with respect to a standard curve and expressed as μg·g^-1^ (proline content/FW). Malonaldehyde (MDA) content was determined following the approach of Heath and Packer [44]. The amount of MDA was expressed as nmol·g (FW).

### Elemental analysis of shoots and roots

Separated roots and shoots were oven-dried (348.15 K, 48 hours) and digested (50 mg/sample) with 4 mL of plasma pure HNO_3_ at 388.15 K. Then the cooled digests were diluted to 25 mL with distilled water. Elemental analysis of Ag was conducted by ICP-MS (Thermo Fisher, ICAP-Qc, USA).

### Statistical analysis

For statistical analysis, one-way analysis of variance (ANOVA) was performed between treatment samples in three replications. Data were analysed by using Spss 26.0. The significant levels of difference for all measured traits were calculated and means were compared by the multiple ranges Duncan test at 5% level. The *P* value smaller or equal to 0.05 was considered as statistically significant.

## Results

### Synthetic process and characterization of AgNPs

**Fig. 1:**
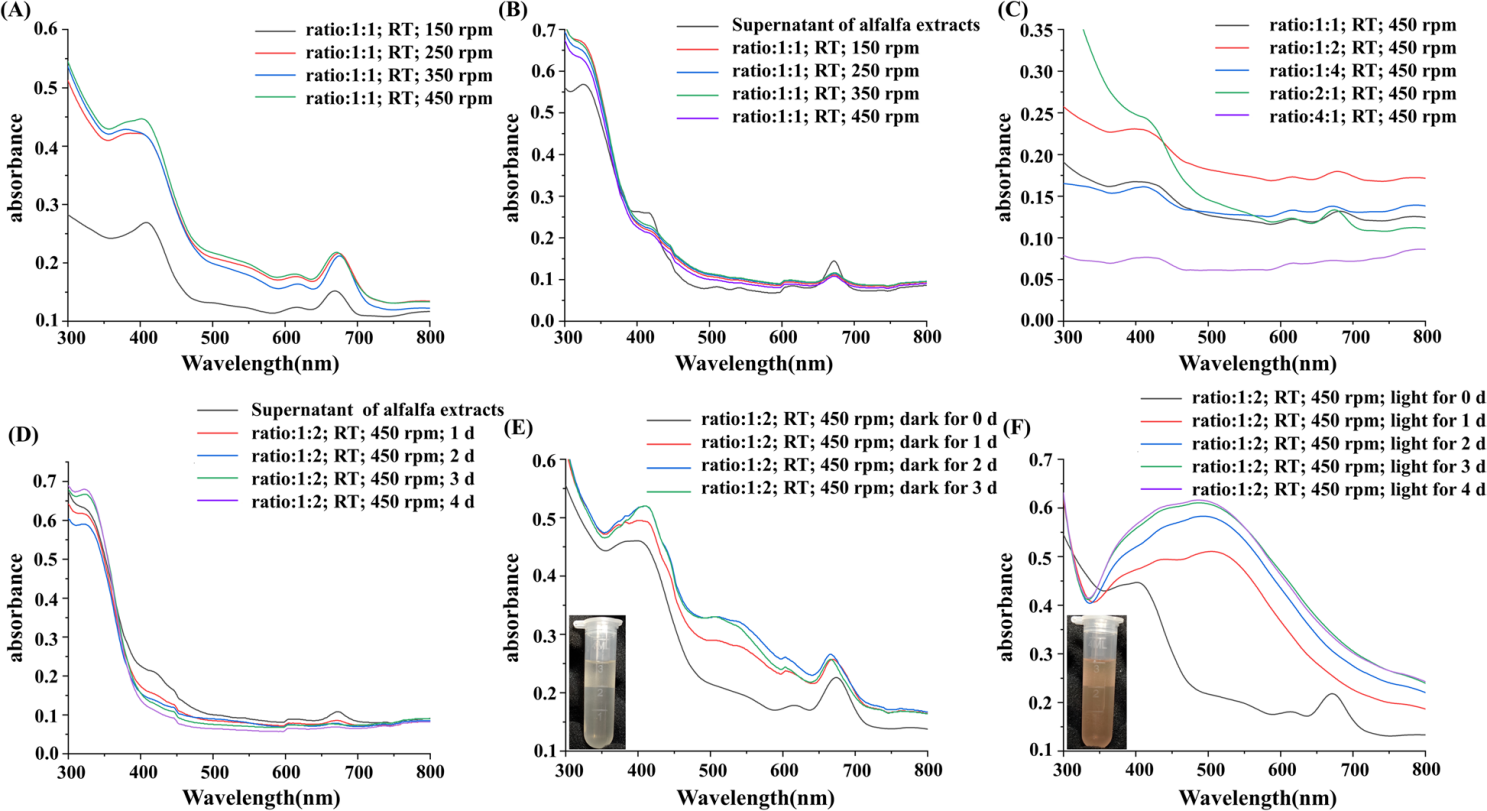
The UV-visible spectra of different reaction conditions. **A** the sediment at different centrifugal rotational speeds; **B** the alfalfa extracts (water: extracts =1:1) and the supernatant at different centrifugal rotational speeds; **C** the sediment at different reactants ratios (2mM AgNO_3_: extracts); **D** the alfalfa extracts (water: extracts=1:2) and the supernatant placed in the dark for different time; **E** the sediment placed in the dark for different time; **F** the sediment placed under the light for different time

In order to optimize the reaction conditions, the centrifugal rotational speed, the reactants ratio, storage time, shading and no shading were considered as variables. We used the intensity of the absorption peak at about 400 nm to measure the concentration of AgNPs (Fig. 1). As for the analysis of synthetic process, not only the absorption peak of generated AgNPs in the sediment, but also the UV-visible absorption spectrum of remaining active substances in the supernatant were measured. In Fig. 1(A) as the centrifugal rotational speed increased from 150 to 450 rpm, the absorption peak became stronger and stronger, which revealed that high speed rotation was beneficial for the synthesis of AgNPs. As seen in Fig. 1(B), the absorption peaks of supernatant decreased with the increase of rotation speed. The ratio of reactants (AgNO_3_: extracts) also had an impact on the formation of AgNPs. The absorption peak of AgNPs at different ratios of reactants was displayed in Fig. 1(C). It was clear that the characteristic absorption peak of AgNPs was the most remarkable at the ratio of 1:2. Moreover, it was also found that the remaining active substances in supernatant were reduced with the increase of standing time, as seen in Fig. 1(D). Overall, a rotation speed of 450 rpm and reactants ratio of 1:2 were the optimal conditions for the synthesis of AgNPs.

If the reactions were carried out in the dark, the colour of synthesized AgNPs was light yellow, as shown in Fig. 1(E). Moreover, the characteristic absorption peak of AgNPs around 400 nm was raised with the increase of standing time, and its intensity was unchanged on the third day. However, if the reactions were conducted in the light, the characteristic absorption peak was red-shifted to about 500 nm and the colour of synthesized AgNPs became pink, as shown in Fig. 1(E). Moreover, under the same rotation speed and the ratio of reactants, the intensity of absorption peak in the light was much higher than that in the dark. So, the preparation conditions of AgNPs were as follows: room temperature, 450 rpm, the reactants ratio (2mM AgNO_3_: extracts) of 1:2, sunlight irradiation for 3 days or dark for 2 days.

**Fig. 2:**
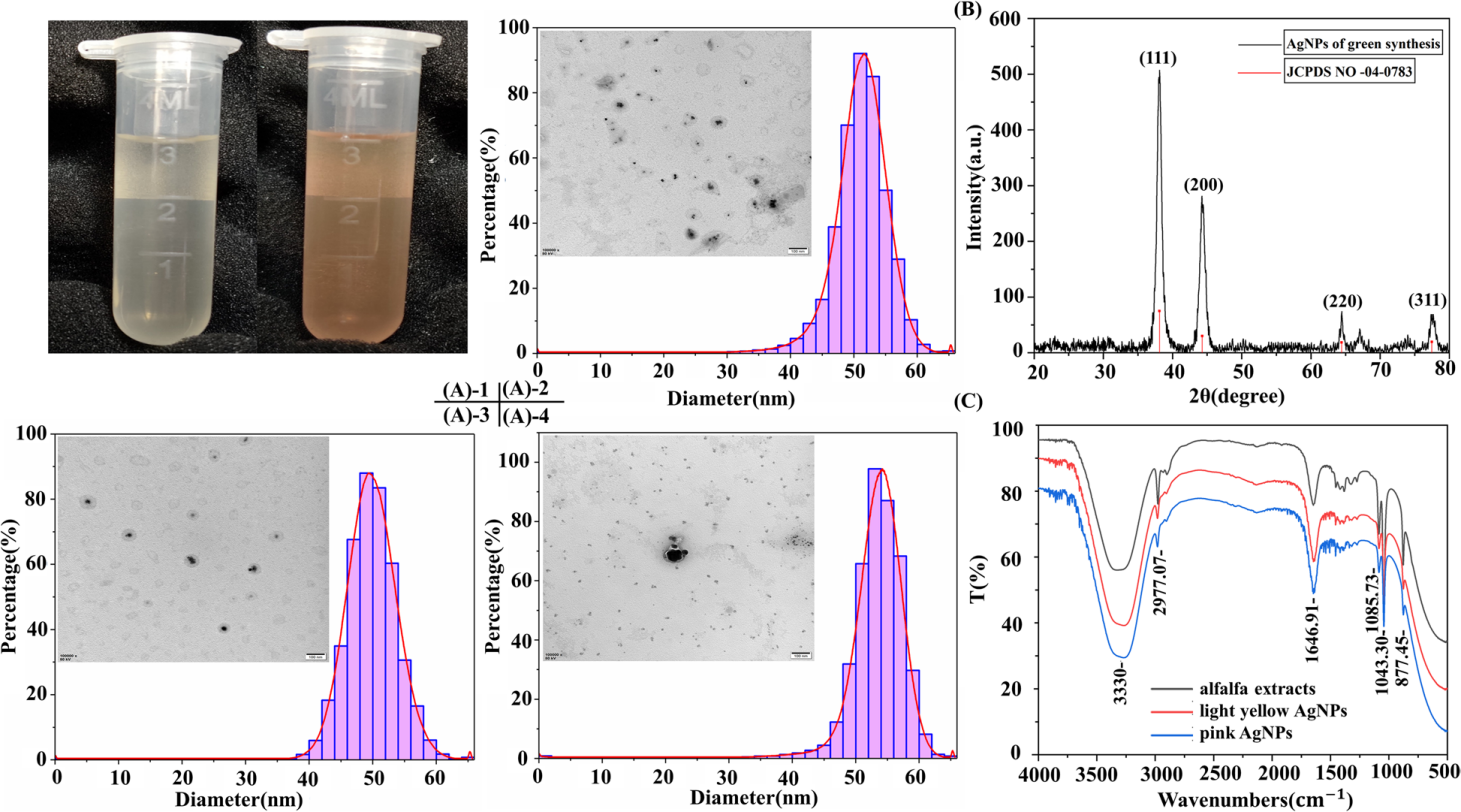
Characterization of green-synthesized AgNPs. (A)-1 light yellow and pink AgNPs solution (10 mg/L); (A)-2 TEM image and particle size distribution map of light yellow AgNPs solution; (A)-3 TEM image and particle size distribution map of pink AgNPs solution; (A)-4 TEM image and particle size distribution map of aggregated particles in pink AgNPs; (B) X-ray diffraction pattern of green-synthesized AgNPs; (C) FTIR spectra of alfalfa extracts and two kinds of AgNPs

**Fig. 3:**
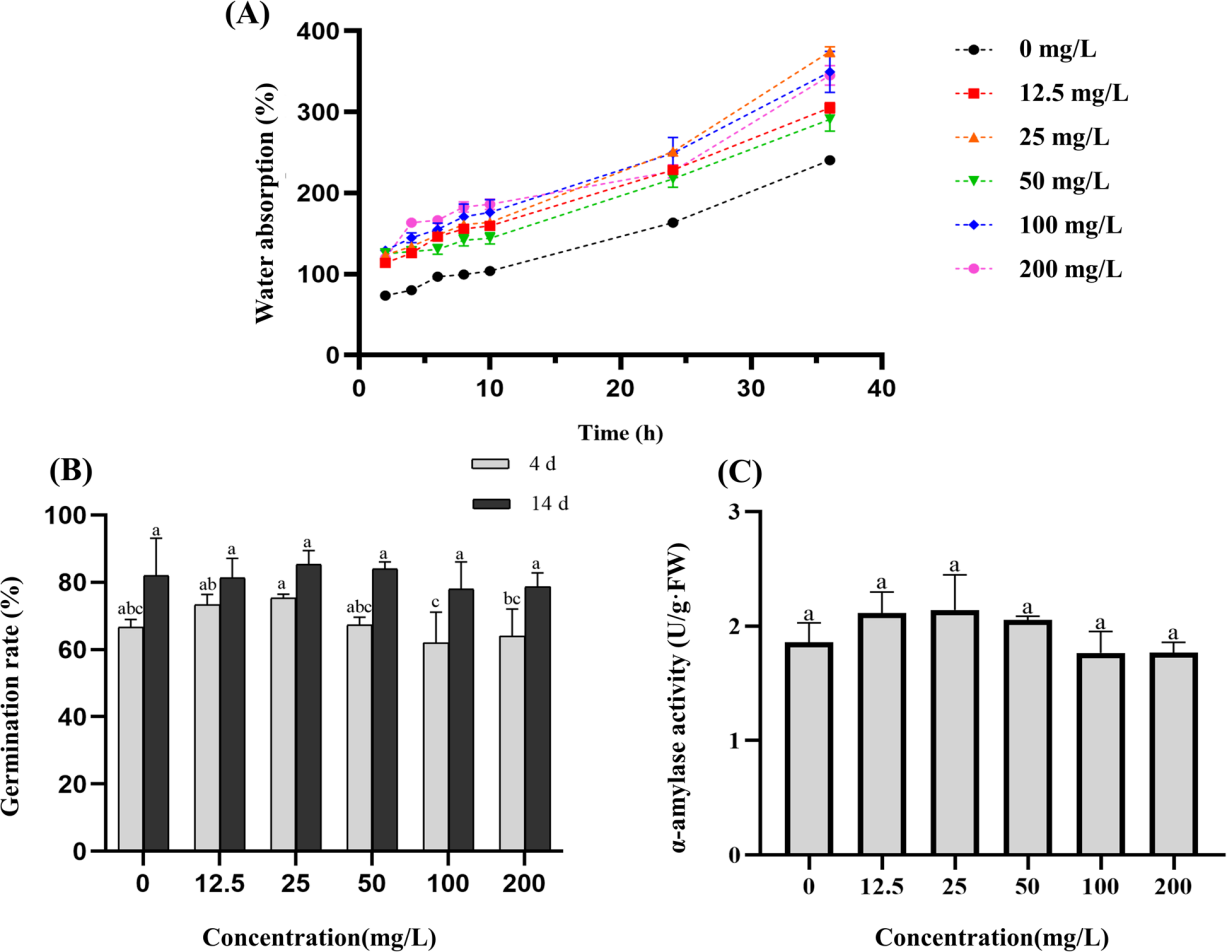
The effect of light-induced AgNPs on seed germination in alfalfa under different concentrations. **A** Water absorption of seeds at different time; **B** Germination rate on the 4 d and 14 d; **C** α-amylase activity

**Fig. 4:**
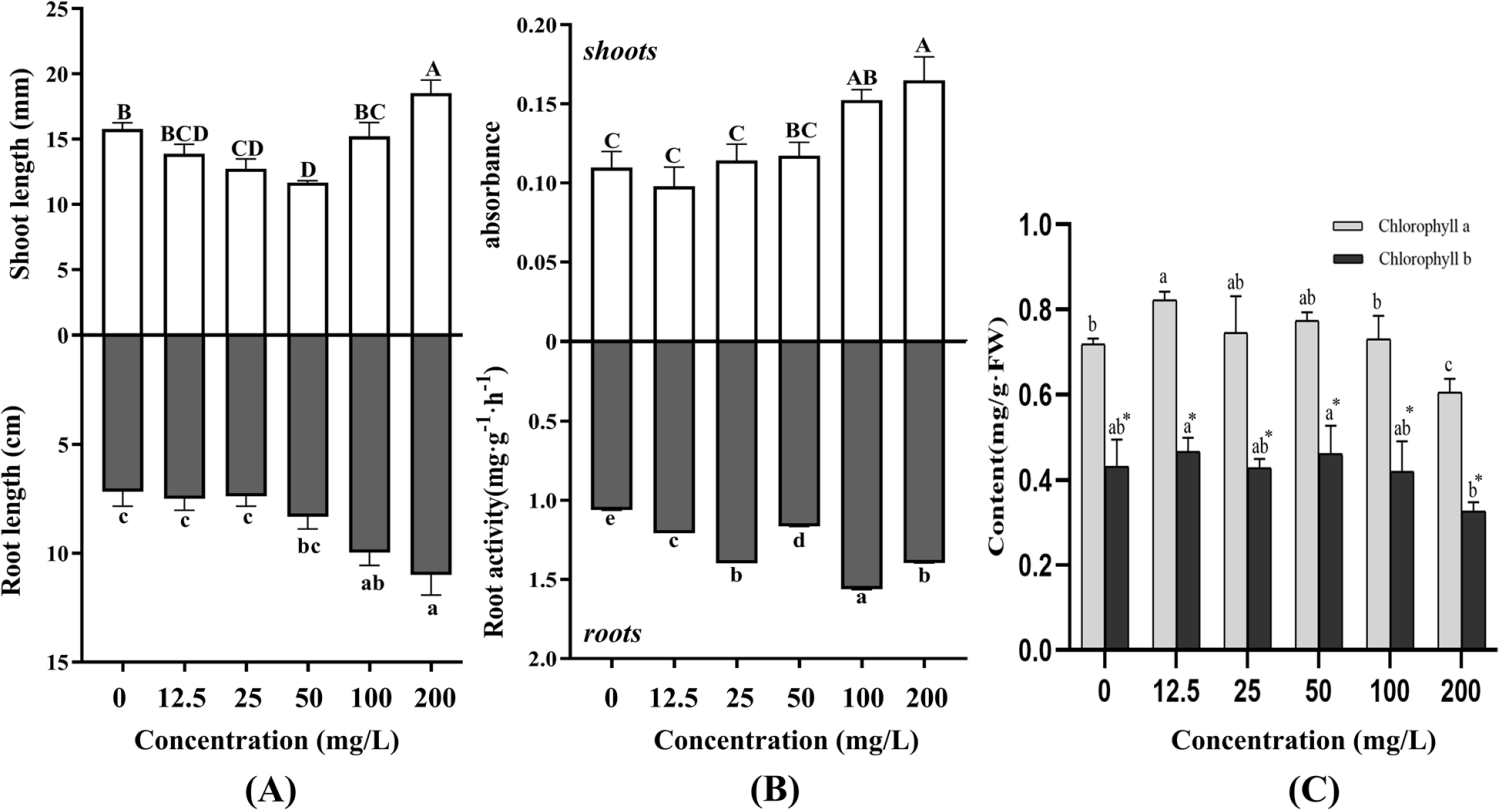
The effects of light-induced AgNPs on seedling growth in alfalfa under different concentrations. **A** Shoot length and root length; **B** Leaves in Evans blue assay and root activity; **C** Chlorophyll a and b content. Different letters show significant differences (*P* < 0.05)

As shown in Fig. 2(A)-1, we synthesized the two colours of AgNPs suspensions. Transmission electron microscopy (TEM) can provide information about shape, particle size and dispersion of NPs. The TEM images and particle size distribution map of the light yellow (Fig. 2(A)-2) and pink AgNPs (Fig. 2(A)-3) indicated that both of them were nearly spherical in shape and their particle sizes were mainly distributed around 50 nm. The size of pink AgNPs was significantly larger than that of light yellow AgNPs owing to aggregation, with some of their particle sizes distributed not only around 50 nm but also around 55 nm (Fig. 2(A)-4). X-ray diffraction (XRD) was adopted to confirm the crystalline nature of the green-synthesized pink AgNPs. In Fig. 2(B), the Bragg Reflection peaks of pink AgNPs were at 38.13°, 44.25°, 64.46° and 77.65° in the 2θ range of 20° to 80°, which can be indexed to the (111), (200), (220) and (311) planes of face-centered cubic (FCC) silver crystal respectively. The sharp diffraction peaks of the pink AgNPs indicated that the pink AgNPs had a good crystallinity. The crystal size of the pink AgNPs was determined to be 39.17 nm by using Debye-Scherrer formula (Additional file 1: Table S1).

In order to identify the functional groups and possible synthesis mechanism, light yellow and pink AgNPs were measured by using Fourier transform infrared (FTIR). In Fig. 2(C), it was observed that all the FTIR spectra had the same shape and six major absorption peaks, which demonstrated that AgNPs were wrapped with the substances of alfalfa extracts. The major absorption peaks at around 3330 cm^-1^, 2977.07 cm^-1^, 1646.91 cm^-1^, 1085.73 cm^-1^, 1043.30 cm^-1^, and 877.45 cm^-1^ were respectively assigned to -OH stretching of phenolic compounds, aromatic -C-H stretching, carboxyl -C=O stretching, ether or ester -C-O-C symmetric stretching, and carboxylic acid -COOH outer curvature. In order to further study the composition of the phytochemicals encapsulated on NPs, high resolution mass spectrometry full scan of primary mass spectrometry plus data-dependent secondary mass spectrometry scan mode (Full MS/dd MS2) was adopted, and the total ion current in the negative and positive ion mode was in Additional file 1: Figure S1. Primary and secondary mass spectra of 20 compounds can be found in Additional file 1: Figure S2. Subsequently, 20 possible compound information can be matched in the database Table 1. Depending on the class of these substances, esters, phenols, carboxylic acids, olefins, sugars and amino acids are all stably present in the AgNPs suspension, and according to the characterisation technique described above, these substances should cover the periphery of the metal core. In addition, these active substances can not only stabilize AgNPs, but also change the toxicity of NPs, especially releasing Ag^+^. It was found that the light-induced AgNPs released much smaller Ag^+^ than light yellow AgNPs in solution. In addition, the concentration of released Ag^+^ was positively correlated with the concentration of AgNPs Table 2. In light-induced AgNPs, the concentration of released Ag^+^ was less than 2 % of the AgNPs concentration. It suggests that the 20 active substances mentioned above form an electrostatic interaction outside the metal nucleus of the nano-silver, stabilising the entire system of the nano-silver suspension and making it less likely that Ag^+^ will be released into the suspension by the AgNPs, thus reducing the amount of free silver ions in the AgNPs suspension.

**Table 1:** Mass parameters for chemical compounds of green-synthesized AgNPs

NO.	RT (min)	m/z	Formula	Name	Molecular Weight
1	0.49	195.05025	C_6_H_12_O_7_	Gluconic acid	196.0575
2	0.55	112.05096	C_4_H_5_N_3_O	Cytosine	111.0437
3	0.55	387.11410	C_12_H_22_O_11_	D- (+)-Maltose	388.1214
4	2.52	186.11258	C_9_H_15_NO_3_	Ecgonine	185.1053
5	7.29	153.05476	C_8_H_8_O_3_	Vanillin	152.0475
6	10.41	195.06540	C_10_H_10_O_4_	Methyl caffeate	194.0581
7	10.63	187.09676	C_9_H_16_O_4_	Azelaic acid	188.1041
8	12.01	201.11258	C_10_H_18_O_4_	3-tert-Butyladipic acid	202.1199
9	12.38	192.13831	C_12_H_17_NO	DEET	191.1310
10	12.86	216.19591	C_12_H_25_NO_2_	12-Aminododecanoic acid	215.1886
11	14.70	231.15904	C_12_H_22_O_4_	Dimethyl sebacate	230.1517
12	15.80	219.17455	C_15_H_22_O	Nootkatone	218.1673
13	16.10	181.04951	C_9_H_8_O_4_	Caffeic acid	180.0421
14	16.39	273.18460	C_18_H_24_O_2_	Galaxolidone	272.1774
15	17.90	243.19624	C_14_H_28_O_3_	2-Hydroxymyristic acid	244.2035
16	18.57	315.25293	C_18_H_34_O_4_	Dibutyl sebacate	314.2454
17	18.70	161.05972	C_10_H_10_O_3_	4-Methoxycinnamic acid	160.0524
18	19.21	256.26334	C_16_H_33_NO	Hexadecanamide	255.2561
19	19.51	309.24231	C_19_H_34_O_4_	Avocadyne 1-acetate	308.2350
20	26.94	106.05021	C_3_H_7_NO_3_	D-serine	105.0429

**Table 2:** Ag^+^ releases from different AgNPs by ICP-OES

Types	0 mg/L	12.5 mg/L	25 mg/L	50 mg/L	100 mg/L	200 mg/L
Pink AgNPs (Light-induced)	-	0.24±0.04^e^	0.48±0.04^d^	0.94±0.06^c^	1.91±0.02^b^	2.75±0.06^a^
Light yellow AgNPs (Dark)	-	3.19±0.06^e^	5.21±0.12^d^	9.87±0.06^c^	19.17±0.06^b^	39.15±0.04^a^

*Values are means of three replicates ± standard deviation; means with different letters are statistically different (Duncan’s multiple comparison at *P* ≤ 0.05)

### Effects of light-induced AgNPs on alfalfa seeds

In Additional file 1: Figure S3, it was observed the presence of Ag in the treated seeds, which indicated that soaking time of 3 hours could cause the silver enrichment. In Additional file 1: Figure S4, it was seen that the seeds of the control group had more cracks on the surface than the treated seeds, while the treated seeds possessed more holes. Moreover, compared to the control group, AgNPs increased intercellular space on the seed surface, and caused cell invagination. Furthermore, it was observed that the seeds formed shrunken epidermis after they were redried, showing a sunken appearance. And there were fewer cracks on the seed coat. These phenomena showed that the treated seeds had a higher water absorption rate than the seeds of control, as shown in Fig. 3(A). In the initial stage (within 10 hours), the water absorption was positively correlated with the concentration of AgNPs. When the germ broke through the seed coats, the water absorption of the more severely shrunken seeds increased more significantly (after 10 hours), especially under the treatment of 25 mg/L AgNPs. Therefore, AgNPs affected the surface of the seed coat and increased the water absorption rate, thereby affecting seed germination. Fig. 3 (B) showed that low concentrations of AgNPs promoted the germination of alfalfa seeds on the 4 d (*P*<0.05), but there was no significant difference in the germination rate on the 14 d. Moreover, green-synthesized AgNPs had no significant effect on α-amylase activity (*P* >0.05) (Fig. 3 (C)).

### Effects of light-induced AgNPs on alfalfa seedlings

In order to further study its effect on alfalfa, important seedling parameters were measured. First, the intuitive and physiological appearance of the seedling was measured. As for shoot length, its value was first decreased and then increased with the increase of AgNPs concentration (*P*<0.05) (Fig. 4(A)). In terms of root length, its value increased with the increase of AgNPs concentration and the roots were more elongated under 50, 100 and 200 mg/L treatments (*P*<0.05) (Fig. 4(A)). On the 7th and 14th days, the roots grew significantly better than shoots (Additional file 1: Figure S5). The treatments not only increased the root length but also strengthened the root activity. Compared with the control, all treatments enhanced the root activity significantly at different amplitudes (Fig. 4(B)).

Furthermore, the leaves were stained with Evans Blue so as to detect the leaf cell death rate. As shown in Fig. 4(B), the cell death in leaves was mainly caused by the high concentrations of AgNPs (*P*<0.05). Under the high concentration of AgNPs, the growth of leaves was obviously worse than the control group (Additional file 1: Figure S5). In Fig. 4(C) the results showed that AgNPs at the low concentrations of 12.5, 25 and 50 mg/L significantly increased chlorophyll a (*P*<0.05), whereas AgNPs at the high concentration of 200 mg/L reduced both chlorophyll a and b (*P*<0.05).

**Fig. 5:**
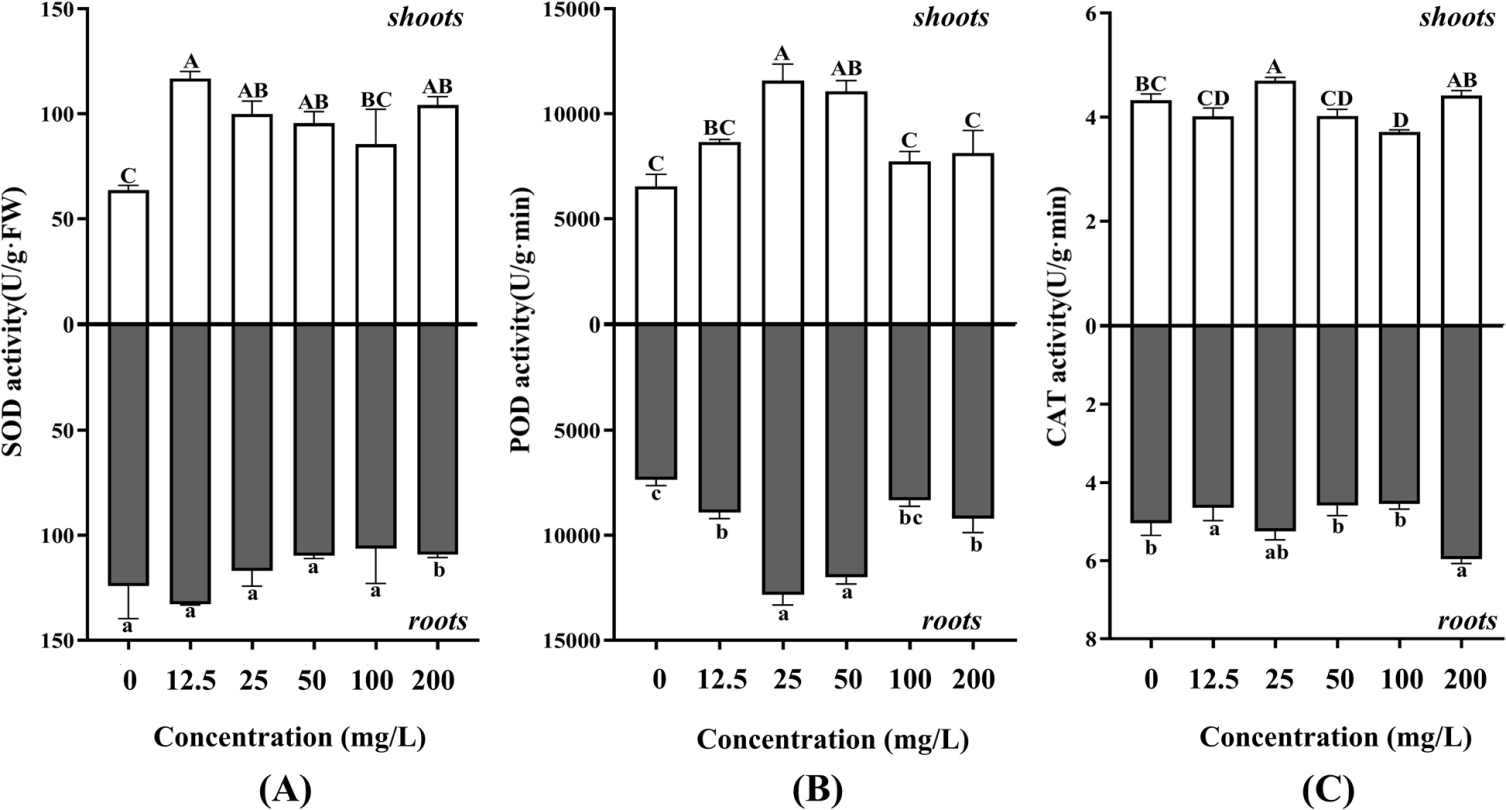
The effects of light-induced AgNPs on antioxidant enzyme system of shoots and roots in alfalfa under different concentrations. **A** SOD activity; **B** POD activity; **C** CAT activity. Different letters show significant differences (*P* < 0.05)

It was found that germination index (GI) and seedling vigor index (SVI I and SVI II) were significantly increased in the nanoprimed alfalfa compared to the control (Additional file 1: Table S2). The FW and DW of the seedlings treated with low concentrations of AgNPs (12.5 mg/L, 25 mg/L, 50 mg/L) were significantly increased (*P*<0.05), while the seedlings treated with high concentration of AgNPs (200 mg/L) were significantly decreased (*P*<0.05). In addition, the antioxidant enzyme system of seedlings was also tested. As shown in Fig. 5, both SOD and POD of antioxidant enzyme systems in shoots and roots were greatly affected by AgNPs (Fig. 5((A) and (B)), and their absorbance presented a trend of first increasing and then decreasing as the AgNPs concentration increased. However, the CAT of antioxidant enzyme system fluctuated slightly under AgNPs treatments (Fig. 5(C)). The antioxidant enzyme activities of roots in all treated seedlings were higher than those of the leaves. In general, the treatment of AgNPs still affected the activity of the antioxidant enzyme system, with SOD enzyme activity having the greatest effect, followed by POD enzyme activity and finally CAT.

**Fig. 6:**
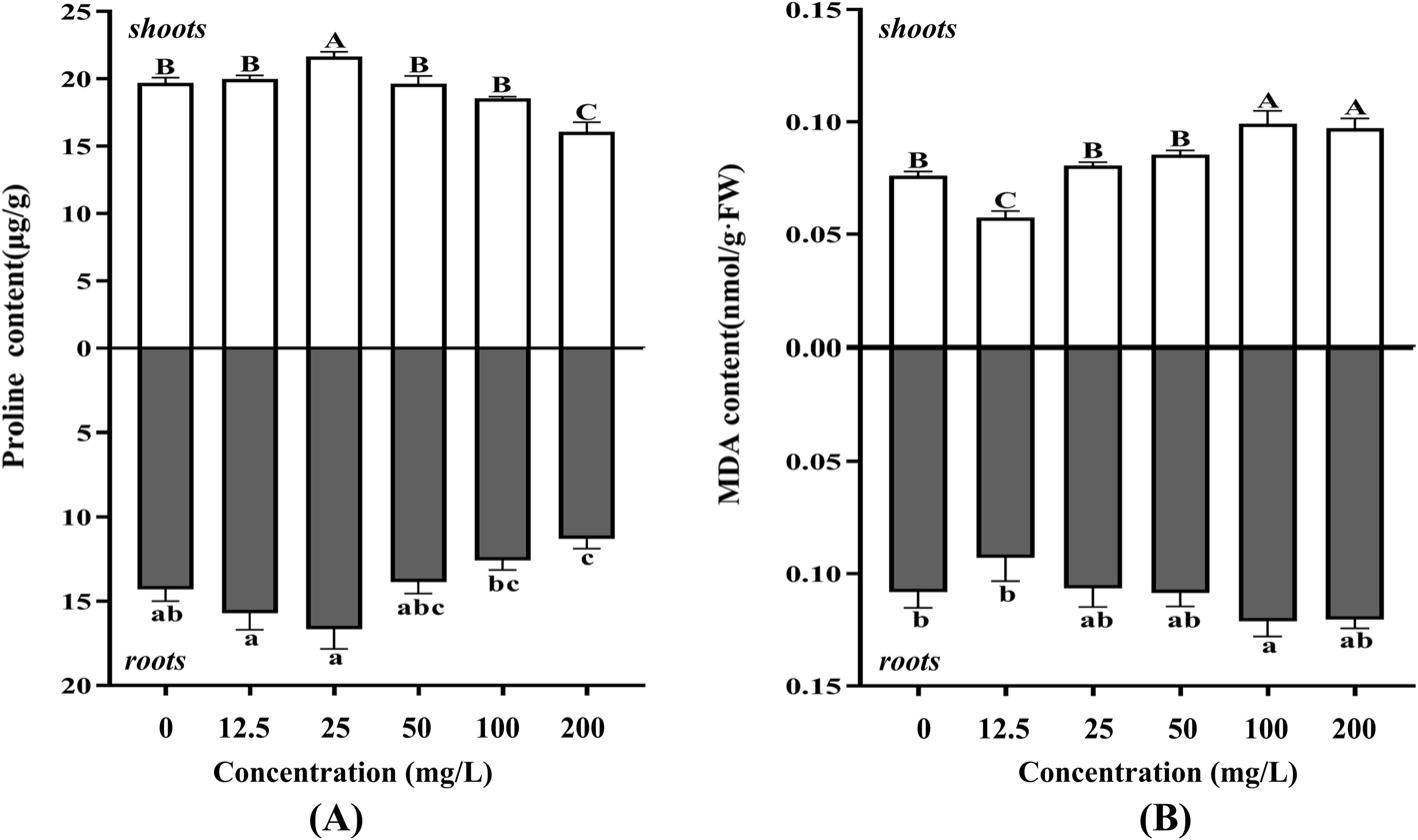
The effects of light-induced AgNPs on proline and MDA contents of shoots and roots in alfalfa under different concentrations. **A** Proline content; **B** MDA content. Different letters show significant differences (*P* < 0.05)

In addition, proline and MDA contents also reflect the important parameters of plants growth. As shown in Fig. 6 (A), proline contents in shoots and roots first increased and then decreased as the AgNPs concentration increased. The proline contents in shoots and roots were only significantly lower than the control group under 200 mg/L AgNPs. As for MDA content, it was found in Fig. 6 (B) that the shoots and roots treated with 12.5 mg/L AgNPs had less MDA accumulation, whereas the groups treated with high concentrations of 100 and 200 mg/L had more MDA than the control. It was showed that the seeds treated with low concentration of AgNPs didn’t damage the membrane and had a greater antioxidant enzyme system to reduce extra reactive oxygen species (ROS). However, although the high concentration treatment also had a greater antioxidant enzyme system than the control group, the membrane was irreversibly damaged. The raw data of all the above physiological experiments were included in Additional file 1: Table S3.

**Fig. 7:**
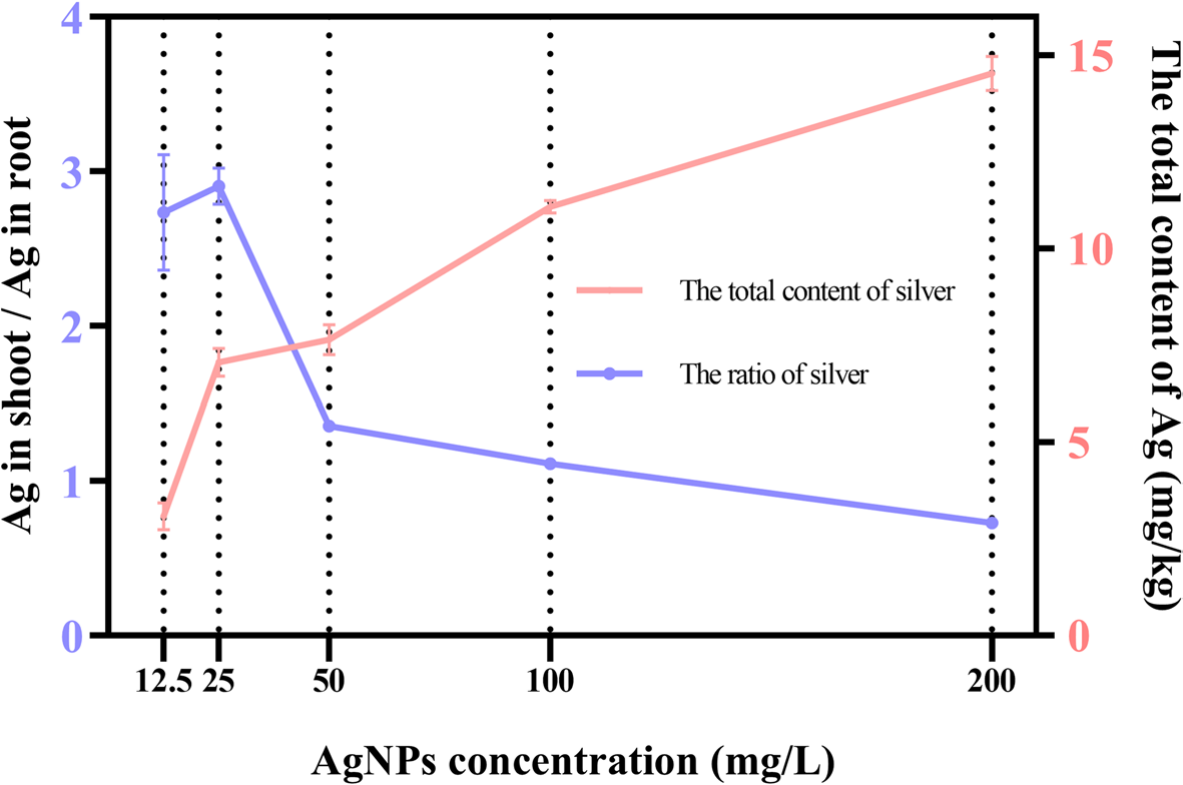
Redistribution of element Ag in shoots and roots under different concentration of AgNPs. Silver content ratio in shoot and root is in purple, and the total content of silver is in pink

Since Ag^+^ was present in seeds under AgNPs treatments, it was necessary to measure the content of Ag^+^ in the shoots and roots of seedlings. With the increase of AgNPs concentration, the content of silver in the seedlings increased and the silver enriched in alfalfa was at the 3-14 mg/kg level (Fig. 7). Moreover, the migration of different concentrations of AgNPs in seedlings was different. Under low concentration AgNPs treatment, the content of silver in shoots was 2-3 times higher than that in roots. Under high concentration AgNPs treatment, the content of silver in roots gradually increased, and finally exceeded the content in shoots. Light-induced AgNPs had a greater impact on shoots at low concentrations, while it had a more important impact on roots at high concentrations.

## Discussion

We used mainly the surface plasmon resonance (SPR) peak around 400 nm in the UV-visible spectrum to determine the reduction of Ag^+^ to AgNPs in the synthesis of AgNPs [45]. In the reaction conditions for the synthesis of AgNPs using alfalfa extracts, we mainly considered the rotational speed and the volume ratio of the reaction material (AgNO_3_: alfalfa extracts) because the temperature affects the composition of the alfalfa extracts, and the placement conditions and time were also found to affect the properties of the AgNPs. Under light placement conditions, aggregation of the AgNPs occurs, resulting in larger particle sizes. As the particles size of the AgNPs becomes larger, the energy required to excite the electrons in the surface plasma is reduced. As a result, in the UV-Vis spectra examined, it can be seen that in the suspension of pink AgNPs, the characteristic peak of AgNPs at 400 nm moves towards the longer wavelength region, i.e. red-shifted, moving between 400-500 nm [46]. Typically, the synthesised AgNPs are yellow in colour and due to the SPR, strong absorption occurs when the frequency of the electromagnetic field resonates with the coherent electron motion, hence the shift in characteristic wavelength and the change in colour in AgNPs suspension [47]. By other characterizations, the shape and size of our synthesized AgNPs are consistent with those synthesized by others at room temperature [1], due to the fact that it is usually at high temperatures that the shape of AgNPs changes, such as cubic, hexagonal, etc. [48], and that more of their own properties require more control over reaction conditions, such as pH and ionic strength etc. The presence of alfalfa extracts within the green synthetic AgNPs can be identified by FTIR, and qualitative analysis yielded 20 phytochemicals in the AgNPs suspension, which theoretically make the AgNPs less toxic than those obtained by chemical synthesis [9].

**Fig. 8:**
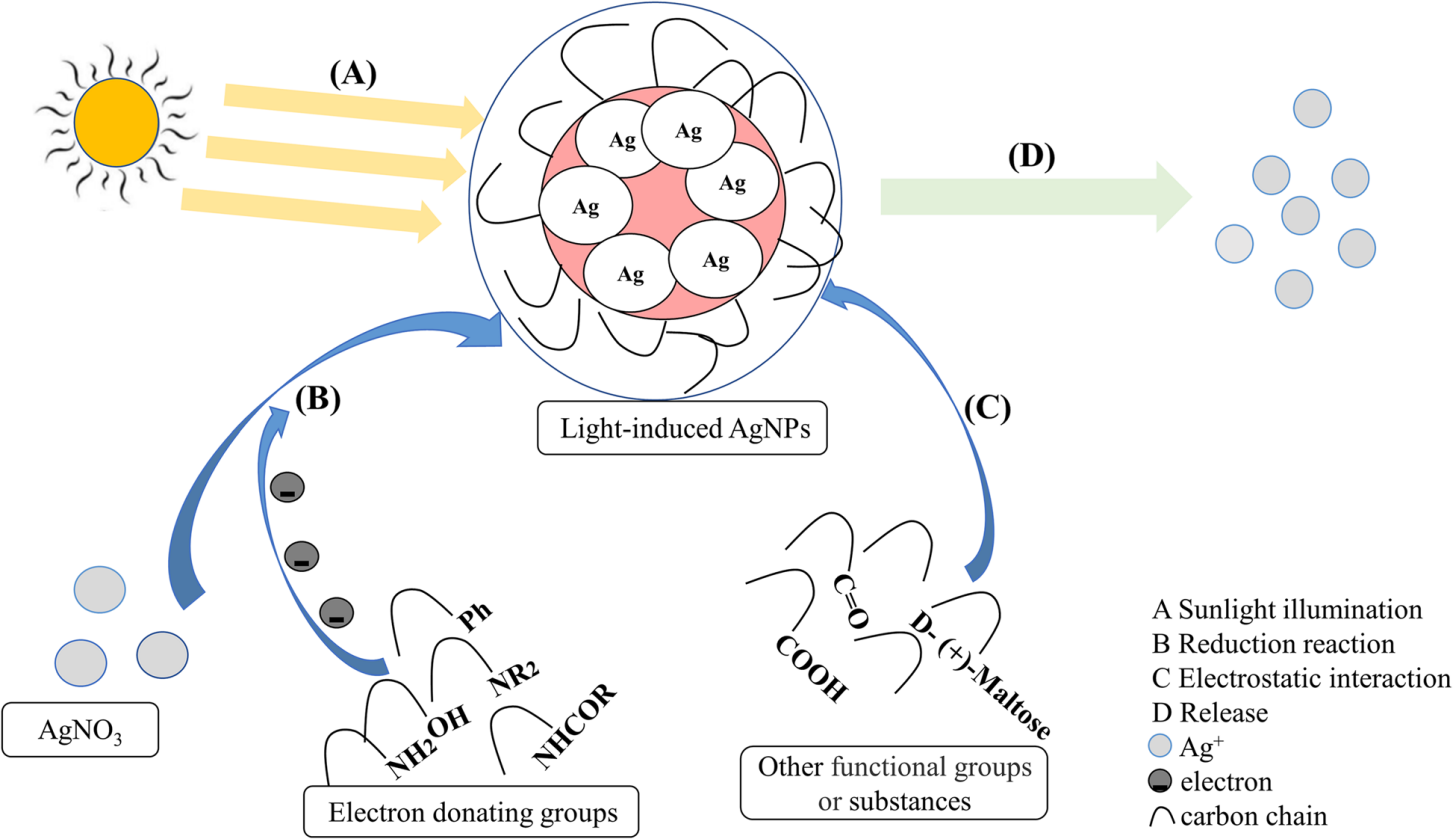
The process of light-induced synthesis of AgNPs and the reduction of its toxicity. **A** Natural light irradiation makes AgNPs aggregated; **B** In the solution, Ag^+^ from AgNO_3_ undergoes a reduction reaction; **C** The remaining substances wrapped the AgNPs through electrostatic interaction; **D** The light-induced AgNPs releases less than 2 % Ag^+^

As shown in Fig. 8, the process of AgNPs synthesis under light placement conditions (Fig. 8 A) may be as follows: of all the reactive substances detected qualitatively, some with the following functional groups have the ability to provide electrons, called electron-giving groups, such as -OH, -NH_2_, -Ph, -NR_2_ and -NHCOR [24], which means that as reactant, Ag^+^ in AgNO_3_ can get enough electrons and undergo a reduction reaction to produce AgNPs (Fig. 8 B). Whereas the remaining detected substances may not have the ability to provide electrons, they can bind to the AgNPs surface through electrostatic interactions and are stabilised by the following functional groups, which allow more active substances to be enriched in the AgNPs periphery, such as functional groups like -COOH, -C=O and maltose (Fig. 8 C) [26]. And these substances seem to act as a protective layer, allowing the AgNPs to release a small amount of free Ag^+^, and it is the small amount of free Ag^+^ that leads to its low biotoxicity (Fig. 8 D) [49]. Since the toxicity of AgNPs is caused by the inherent properties of AgNPs, which can be reduced by the light reaction [50], in the ICP-OES measurement of the Ag^+^ content, we obtained that both placements of AgNPs suspensions had less free Ag^+^, except that the pink AgNPs suspension for the light placement condition had less free Ag^+^. It would suggest that the AgNPs suspension for the light placement condition not only contains phytochemicals from alfalfa, but also has a very small amount of free Ag^+^ and would therefore be less toxic in vivo and in vitro to the organism than the chemically synthesized NPs [30]. Normally, metal NPs suspensions are known to have some free metal ions, but if the appropriate synthesis is applied, it may stimulate resistance in plants [26], in which case it is of interest to study the interaction between AgNPs and plants [22].

Unlike other elements, silver is not an essential nutrient element for plants, so it cannot be considered as a fertilizer. Short-term exposure to NPs, the accumulation of NPs with priming was much lower than direct spraying or hydroponics [33]. And this exposure can form pores on the seeds, thereby increasing the water absorption rate of seeds [24]. Because of this physical change, the seed germination rate had changed, but it had not been observed that the α-amylase activity described by others was the dominant cause [25]. These small holes allowed a large amount of AgNPs and water to enter, making it easier for the seeds to germinate [24]. Through physiological indicators and silver element analysis, the distribution of silver was responsible for the physiological indicators of plants. The ROS in plants was in a dynamic balance under normal conditions, but the equilibrium state will be destroyed under adversity. Subsequently, it would lead to the accumulation of ROS. Therefore, in order to survive, plants would enhance the activity of antioxidant enzyme system to remove excess ROS [51]. As AgNPs entered the seeds, it promoted the accumulation of silver in the shoots and roots, which led to varying degrees of response of the antioxidant enzyme system. Under low concentration, compared with roots, silver was mainly concentrated in the shoots, and had a slightly positive effect on the photosynthesis. It would be nontoxic to the leaf cells and there was no elongation effect on shoots. However, our founding was in conflict with the result that low concentration AgNPs was also toxic to plants [52]. It was because Ag^+^ forms complexes with several essential intracellular biomolecules that can disrupt the normal physiology and growth of seedlings [53, 54]. In contrast, the dissolution of AgNPs into Ag^+^ depends on a number of important physicochemical parameters such as size, concentration, pH, temperature, ionic strength and the presence or absence of ligands [55]. Whereas green synthetic AgNPs is stabilised by reactive substances [56], these reactive substances influence the dissolution of AgNPs into Ag^+^ and reduce the release of Ag^+^ [57]. Under high concentration, compared with shoots, silver was mainly concentrated in the roots, and had a certain positive effect on root cell division [58], increasing root activity and elongation [59]. In addition, when the silver distribution was in a balanced state, the DW and FW of the seedlings can reach the maximum. It was because the concentration of NPs had an impact on plants, and plants consumed nutrients too fast under low-dose treatments, resulting in some positive effects that were not fully amplified on the 14th day; and under high-dose treatments, although plants had some positive effects, it showed a slight toxic effect because of the accumulation of silver. The NPs with lower surface areas can worse interfere with cell membrane function by directly reacting with the membrane [60]. Moreover, under low-concentration treatment, proline accumulated in the roots due to the increase of enzyme activity, thereby absorbing a lot of water and decomposing the stored nutrients. However, since only water was provided in the Petri dishes, which led to premature consumption of nutrients and enhanced photosynthesis to produce nutrients. As the concentration of AgNPs increased, seedlings showed a certain damage. Thus, the distribution of Ag^+^ explained the effects of AgNPs on the physiological of seedlings and roots under above-mentioned different concentrations [61]. Furthermore, the intake of silver by the seedlings is limited and excess silver can cause irreversible damage to the leaves of the seedlings [62].

Of course, with AgNPs and Ag^+^, it cannot say who is the main factor affecting the alfalfa. It is because the roots of plants can take up AgNPs directly [63], or they can reduce Ag^+^ to AgNPs in vivo via reducing sugars and other antioxidants in the roots [64]. Therefore, the reaction of the AgNPs suspensions with the free Ag^+^ is complex in plants. It was shown that the proportion of Ag^+^ in shoots was higher than in roots because of the oxidation that occurred in the shoots [65]. Overall, there is an interconversion between AgNPs and Ag^+^ in plants, with AgNPs acting as a carrier and source of Ag^+^. In addition, due to the extensive bactericidal effect of AgNPs, and the fact that seed endophytic bacteria may control germination and seedling establishment. So, it is necessary to do more subsequent studies of nanosilver against seed endophytic bacteria [66].

## Conclusion

In general, we used ultrasonic-assisted ethanol extraction to obtain the alfalfa extracts that were rich in many active substances. After that, we successfully synthesized light-induced AgNPs by using the alfalfa extracts. Before characterizing, we optimized the reaction conditions by considering, the centrifugal rotational speed, the reactants ratio, storage time, shading and no shading as variables. It was found that light-induced AgNPs released less Ag^+^ in solution because of being wrapped with esters, phenols, acids, terpenes, amino acid, sugars and so on. Applying light-induced AgNPs to alfalfa, we can find that the toxicity of AgNPs was related to its concentration and the distribution of silver in shoots and roots. In addition, nanopriming promoted early germination of seeds by increasing the water absorption rate of seeds. There was a certain toxicity in AgNPs because of accumulation, but their toxicity can be reduced by using reasonable synthesis and application methods.

In a word, if the engineered particles are properly coated and synthesized by light induction, NPs with lower toxicity would be obtained. Using nanopriming can reduce the minimum exposure of plants to metals, and can have unlimited positive effects on plants, providing more possibilities for nano-agriculture.

## Supplementary Information

**Additional file 1.** The total ion current in the negative and positive ion mode in light-induced AgNPs (Figure S1); Primary and secondary mass spectra of 20 chemical substances detected in green-synthesized AgNPs (Figure S2); SEM and EDS patterns of seeds cross-sections treated with different concentrations (Figure S3); SEM images of seeds (Figure S4); Influence of different nanopriming treatment on alfalfa seed in petri dishes (Figure S5); Calculation of pink AgNPs’ crystal size by using Debye-Scherrer formula (Table S1); Effect of AgNPs nanopriming on growth parameters of 14 d seedlings (Table S2); Raw data for all growth parameters of 14 d seedlings under AgNPs treatments (Table S3).

## Data Availability

All the datasets generated and analysed during the current study were uploaded as with the manuscript as additional files. Primary and processed data are available in additional file.
