# Supplementary material for: Green nanopriming: responses of alfalfa (Medicago sativa L.) seedlings to alfalfa extracts capped and light-induced silver nanoparticles
Source: BMC Plant Biol. 2022 Jul 5;22:323. doi: 10.1186/s12870-022-03692-9 (PMC9254587; doi:10.1186/s12870-022-03692-9)
Supplement: Supplementary file 1 — Additional file 1. The total ion current in the negative and positive ion mode in light-induced AgNPs (Figure S1); Primary and secondary mass spectra of 20 chemical substances detected in green-synthesized AgNPs (Figure S2); SEM and EDS patterns of seeds cross-sections treated with different concentrations (Figure S3); SEM images of seeds (Figure S4); Influence of different nanopriming treatment on alfalfa seed in petri dishes (Figure S5); Calculation of pink AgNPs’ crystal size by using Debye-Scherrer formula (Table S1); Effect of AgNPs nanopriming on growth parameters of 14 d seedlings (Table S2); Raw data for all growth parameters of 14 d seedlings under AgNPs treatments (Table S3). [file 12870_2022_3692_MOESM1_ESM.docx]

Supplementary Information

**Green nanopriming: Responses of alfalfa (*Medicago sativa* L.) seedlings to alfalfa extracts capped and light-induced silver nanoparticles**

Kexiao Song,^†^ Donghao Zhao,^†^ Haoyang Sun,^†^ Jinzhu Gao,^†^ Shuo Li,^†^ Tianming Hu,^*,†^ Xueqing He^*,†^

^1^College of Grassland Agriculture, Northwest A&F University, Yangling, Shaanxi Province, China

* Corresponding author: Xueqing He (E-mail: [hexueqing@nwsuaf.edu.cn](mailto:hexueqing@nwsuaf.edu.cn));

Tianming Hu (E-mail: hutianming@126.com).

Number of pages: 25

Number of tables: 3

Number of figures: 5

Figure S1. The total ion current in the negative (black) and positive (red) ion mode

Figure S2. Primary and secondary mass spectra of 20 chemical substances detected in green-synthesized AgNPs

Figure S3. SEM and EDS images of seeds cross-sections treated with different concentrations AgNPs. (The left column is a cross-section of the treated seed, where the white boxed area is the EDS spectrometer scan area; the right column is the elemental result of the scan)

**
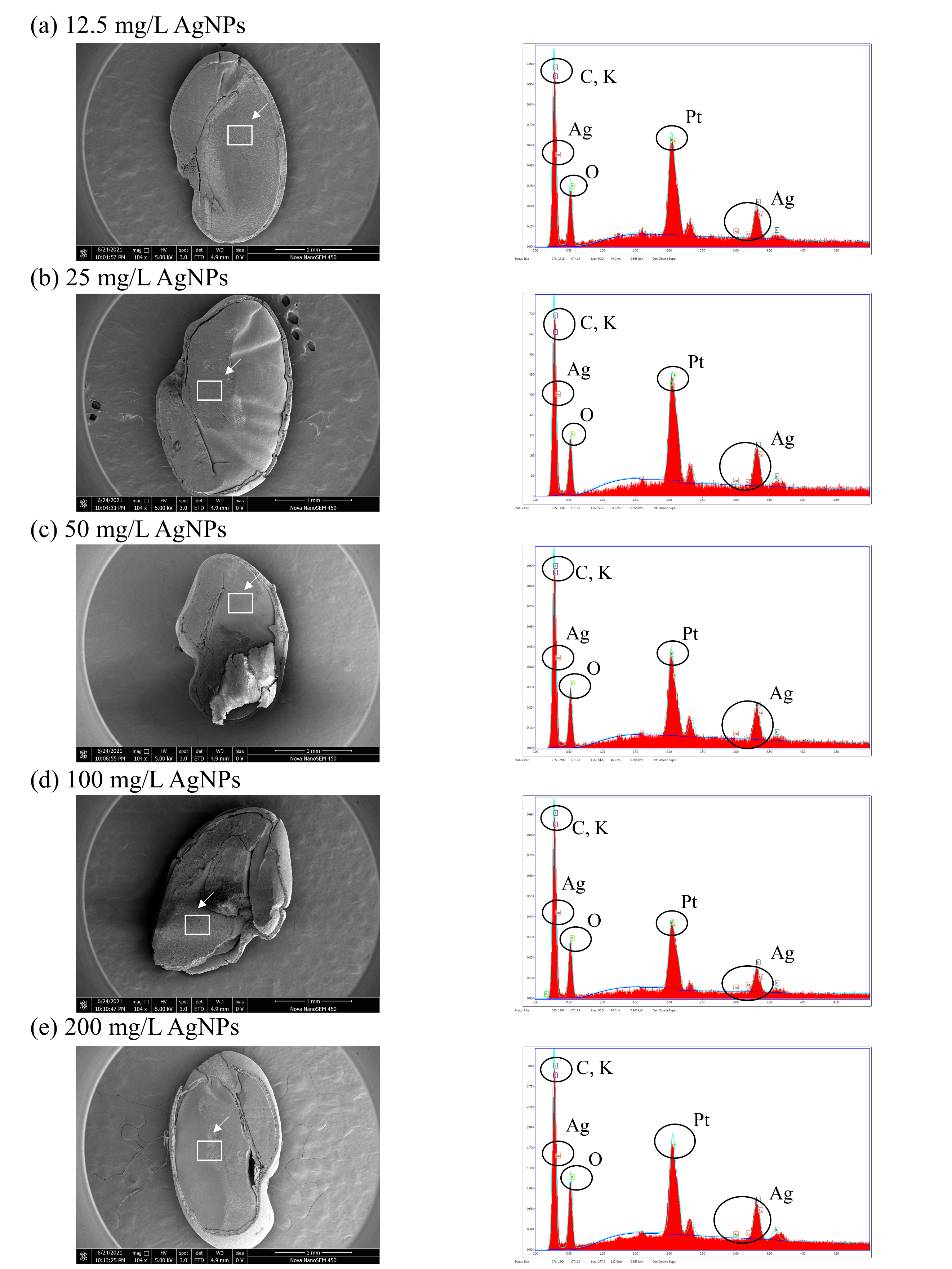
**

Figure S4. SEM images of seed coat treated with different concentrations AgNPs. (A,B-1) 0 mg/L AgNPs; (A,B-2) 12.5 mg/L AgNPs; (A,B-3) 25 mg/L AgNPs; (A,B-4) 50 mg/L AgNPs; (A,B-5) 100 mg/L AgNPs; (A,B-6) 200 mg/L AgNPs. (A is an overall view of the seeds; B is a partial enlargement of the seed coat)


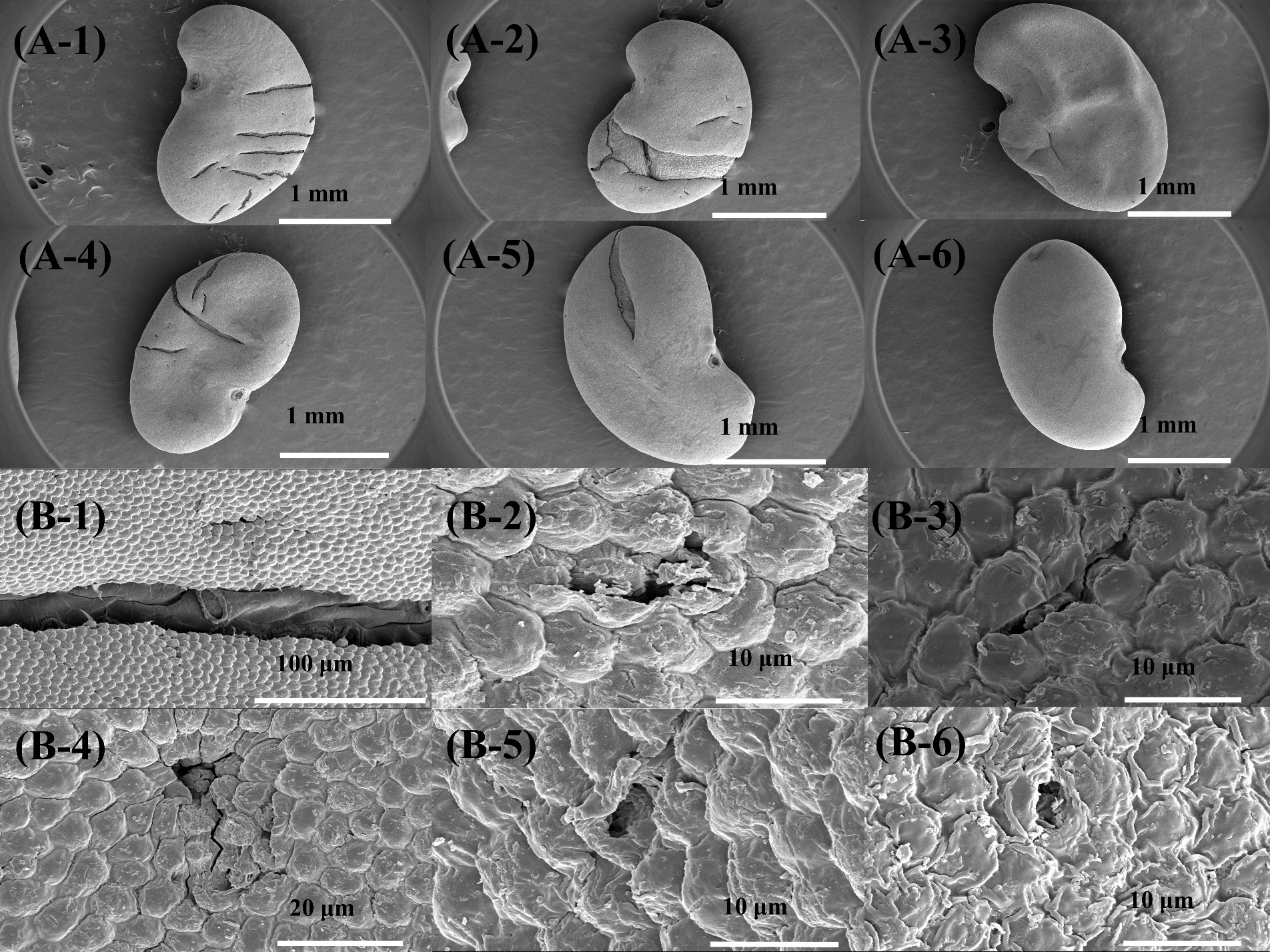


Figure S5. Influence of different nanopriming treatment on alfalfa seed in petri dishes.


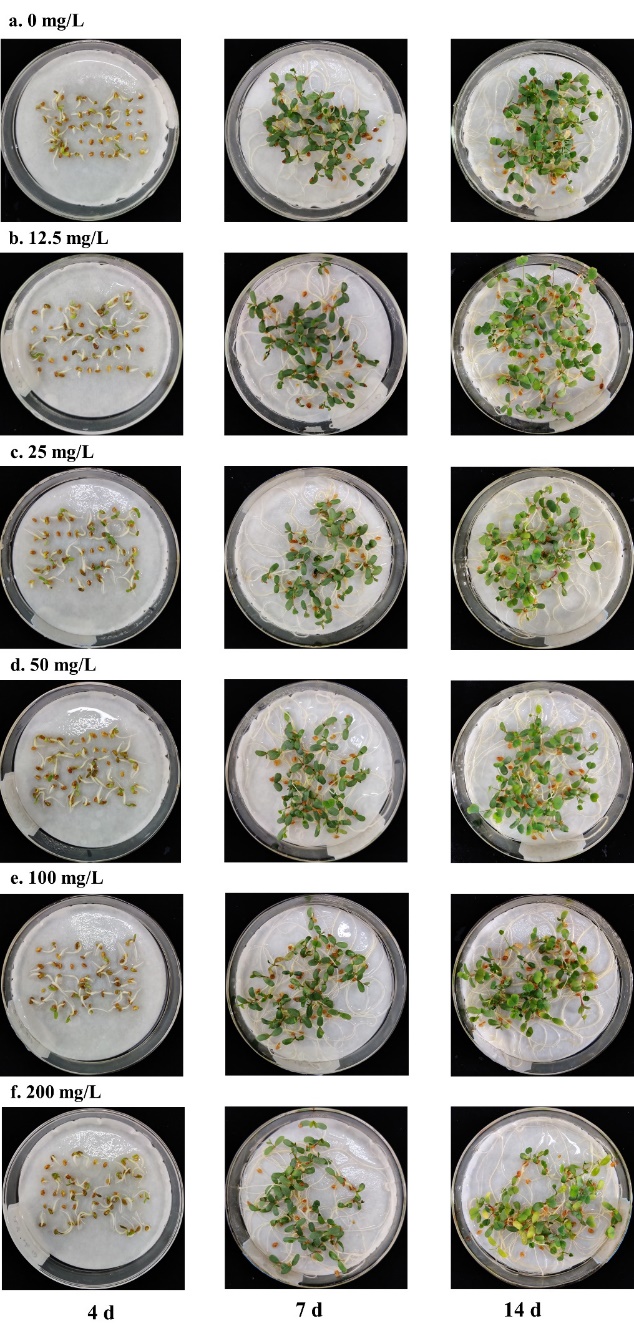


Table S1. Calculation of pink AgNPs’ crystal size by using Debye-Scherrer formula.

| (hkl) | K | λ/nm | 2θ/° | FWHM | D/nm |
| --- | --- | --- | --- | --- | --- |
| (111) | 1 | 0.15405 | 38.13 | 0.747 | 15.03 |
| (200) | 1 | 0.15405 | 44.25 | 0.762 | 16.18 |
| (220) | 1 | 0.15405 | 64.46 | 0.389 | 52.65 |
| (311) | 1 | 0.15405 | 77.65 | 0.567 | 72.82 |
| Average | - | - | - | - | 39.17 |

Table S2. Effect of AgNPs nanopriming on growth parameters of 14 d seedlings.

| Parameter | 0 mg/L | 12.5 mg/L | 25 mg/L | 50 mg/L | 100 mg/L | 200 mg/L |
| --- | --- | --- | --- | --- | --- | --- |
| Relative root elongation (RRE)/% | - | 104.51±12.72^d^ | 102.94±10.96^e^ | 118.28±13.40^c^ | 139.08±13.99^b^ | 153.28±22.48^a^ |
| Germination index (GI)/% | - | 103.53±12.62^e^ | 106.94±9.87^d^ | 121.38±16.60^c^ | 131.69±10.72^b^ | 147.77±28.86^a^ |
| Seedling vigor index (SVI I) | 7.10±0.34^f^ | 7.21±0.83^e^ | 7.37±0.67^d^ | 7.89±1.03^c^ | 8.92±0.68^b^ | 10.15±1.85^a^ |
| Seedling vigor index (SVI II) | 0.30±0.04^e^ | 0.33±0.04^d^ | 0.36±0.02^c^ | 0.39±0.02^a^ | 0.38±0.03^b^ | 0.38±0.03^b^ |
| Fresh weight (FW)/g | 1.45±0.01^d^ | 1.76±0.02^a^ | 1.60±0.02^b^ | 1.57±0.01^c^ | 1.44±0.01^d^ | 1.28±0.01^e^ |
| Dry weight (DW)/g | 0.35±0.01^c^ | 0.48±0.01^a^ | 0.42±0.01^b^ | 0.37±0.01^c^ | 0.33±0.01^d^ | 0.29±0.01^e^ |

*Values are means of three replicates ± standard deviation; means with different letters are statistically different (Duncan’s multiple comparison at *P* ≤ 0.05).

Table S3. Effect of nanopriming with increasing concentration of AgNPs on growth parameters of 14 d seedlings.

1. Fresh weight (FW) and Dry weight (DW)

| Weight/g | Treatments | | | | | |
| --- | --- | --- | --- | --- | --- | --- |
|  | 0 mg/L | 12.5 mg/L | 25 mg/L | 50 mg/L | 100 mg/L | 200 mg/L |
| Fresh weight (FW)/g | 1.435  1.452  1.456 | 1.743  1.754  1.777 | 1.612  1.598  1.582 | 1.572  1.568  1.555 | 1.440  1.452  1.432 | 1.265  1.279  1.282 |
| Dry weight (DW)/g | 0.345  0.354  0.359 | 0.476  0.482  0.471 | 0.421  0.419  0.411 | 0.372  0.369  0.360 | 0.342  0.321  0.329 | 0.301  0.291  0.287 |

1. Relative root elongation (RRE)/%

| Treatments | (1)/% | (2)/% | (3)/% | Average/% |
| --- | --- | --- | --- | --- |
| 12.5 mg/L | 118.54 | 93.72 | 101.28 | 104.51 |
| 25 mg/L | 115.26 | 94.28 | 99.27 | 102.94 |
| 50 mg/L | 104.60 | 118.85 | 131.38 | 118.28 |
| 100 mg/L | 154.45 | 135.70 | 127.09 | 139.08 |
| 200 mg/L | 176.46 | 131.58 | 151.80 | 153.28 |

1. Relative seed germination/%

| Treatments | (1)/% | (2)/% | (3)/% | Average/% |
| --- | --- | --- | --- | --- |
| 12.5 mg/L | 95.12 | 95.12 | 107.32 | 99.19 |
| 25 mg/L | 102.44 | 109.76 | 100.00 | 104.07 |
| 50 mg/L | 100.00 | 102.44 | 104.88 | 102.44 |
| 100 mg/L | 85.37 | 104.88 | 95.12 | 95.12 |
| 200 mg/L | 100.00 | 90.24 | 97.56 | 95.93 |

1. Germination index (GI)/%

| Treatments | (1)/% | (2)/% | (3)/% | Average/% |
| --- | --- | --- | --- | --- |
| 12.5 mg/L | 112.76 | 89.15 | 108.69 | 103.53 |
| 25 mg/L | 118.07 | 103.48 | 99.27 | 106.94 |
| 50 mg/L | 104.60 | 121.75 | 137.79 | 121.38 |
| 100 mg/L | 131.85 | 142.32 | 120.89 | 131.69 |
| 200 mg/L | 176.46 | 118.74 | 148.10 | 147.77 |

1. Seedling vigor index (SVI I)

| Treatments | (1) | (2) | (3) | Average |
| --- | --- | --- | --- | --- |
| 0 mg/L | 7.29 | 6.70 | 7.28 | 7.10 |
| 12.5 mg/L | 7.83 | 6.27 | 7.55 | 7.21 |
| 25 mg/L | 8.13 | 7.13 | 6.85 | 7.37 |
| 50 mg/L | 7.09 | 7.74 | 9.10 | 7.98 |
| 100 mg/L | 8.97 | 9.58 | 8.22 | 8.92 |
| 200 mg/L | 12.04 | 8.35 | 10.05 | 10.15 |

1. Seedling vigor index (SVI II)

| Treatments | (1) | (2) | (3) | Average |
| --- | --- | --- | --- | --- |
| 0 mg/L | 0.2484 | 0.2832 | 0.3375 | 0.2897 |
| 12.5 mg/L | 0.2832 | 0.3375 | 0.3713 | 0.3307 |
| 25 mg/L | 0.3375 | 0.3713 | 0.3760 | 0.3616 |
| 50 mg/L | 0.3713 | 0.3760 | 0.4145 | 0.3872 |
| 100 mg/L | 0.3760 | 0.4145 | 0.3536 | 0.3813 |
| 200 mg/L | 0.4144 | 0.3536 | 0.3771 | 0.3817 |

1. Germination rate in 4 d and 14 d

| Treatments | Germination rate (%) | |
| --- | --- | --- |
|  | 4 d | 14 d |
| 0 mg/L | 68/68/64 | 72/80/94 |
| 12.5 mg/L | 70/74/76 | 78/78/88 |
| 25 mg/L | 76/76/74 | 84/90/82 |
| 50 mg/L | 66/70/66 | 82/84/86 |
| 100 mg/L | 54/72/60 | 70/86/78 |
| 200 mg/L | 56/64/72 | 82/74/80 |

1. α-amylase activity

| Treatments | Weight/g | Absorbance  (540 nm) | α-amylase activity (U/g·FW) |
| --- | --- | --- | --- |
| 0 mg/L-1 | 0.1822 | 0.191 | 1.617149 |
| 0 mg/L-2 | 0.1354 | 0.192 | 2.185764 |
| 0 mg/L-3 | 0.1668 | 0.192 | 1.774295 |
| 12.5 mg/L-1 | 0.1620 | 0.269 | 2.448470 |
| 12.5 mg/L-2 | 0.1417 | 0.191 | 2.079355 |
| 12.5 mg/L-3 | 0.1632 | 0.192 | 1.813434 |
| 25 mg/L-1 | 0.1696 | 0.195 | 1.768135 |
| 25 mg/L-2 | 0.1598 | 0.198 | 1.901120 |
| 25 mg/L-3 | 0.1089 | 0.195 | 2.753680 |
| 50 mg/L-1 | 0.1455 | 0.199 | 2.096954 |
| 50 mg/L-2 | 0.1420 | 0.191 | 2.074962 |
| 50 mg/L-3 | 0.1489 | 0.192 | 1.987591 |
| 100 mg/L-1 | 0.1782 | 0.195 | 1.682804 |
| 100 mg/L-2 | 0.2009 | 0.195 | 1.492662 |
| 100 mg/L-3 | 0.1423 | 0.197 | 2.125730 |
| 200 mg/L-1 | 0.1584 | 0.194 | 1.884899 |
| 200 mg/L-2 | 0.1868 | 0.193 | 1.591329 |
| 200 mg/L-3 | 0.1612 | 0.191 | 1.827820 |

1. Shoot length

| Treatments | Shoot length (mm) | | | | | |
| --- | --- | --- | --- | --- | --- | --- |
|  | 1 | 2 | 3 | 4 | 5 | Average |
| 0 mg/L-1 | 16.11 | 18.91 | 15.4 | 17.26 | 15.32 | 16.6 |
| 0 mg/L-2 | 13.26 | 11.52 | 18.63 | 19.35 | 16.31 | 15.814 |
| 0 mg/L-3 | 14.83 | 12.16 | 14.21 | 17.76 | 15.58 | 14.908 |
| 12.5 mg/L-1 | 16.01 | 9.71 | 16.34 | 20.65 | 14.02 | 15.346 |
| 12.5 mg/L-2 | 13.86 | 13.81 | 11.12 | 13.43 | 13.68 | 13.18 |
| 12.5 mg/L-3 | 11.46 | 11.64 | 10.6 | 18.07 | 13.97 | 13.148 |
| 25 mg/L-1 | 8.17 | 14.37 | 15.7 | 15.28 | 17.45 | 14.194 |
| 25 mg/L-2 | 13.66 | 12.22 | 12.22 | 10.87 | 8.98 | 11.59 |
| 25 mg/L-3 | 17.22 | 10.58 | 8.33 | 10 | 15.89 | 12.404 |
| 50 mg/L-1 | 12.81 | 10.15 | 10.15 | 12.03 | 12.03 | 11.434 |
| 50 mg/L-2 | 12.35 | 8.41 | 13.69 | 13.69 | 11.71 | 11.97 |
| 50 mg/L-3 | 8.26 | 15.76 | 11.89 | 9.57 | 12.58 | 11.612 |
| 100 mg/L-1 | 18.37 | 12.01 | 24.33 | 14.84 | 17.21 | 17.352 |
| 100 mg/L-2 | 8.54 | 12.25 | 19.13 | 20.18 | 10.15 | 14.05 |
| 100 mg/L-3 | 14.55 | 18.48 | 19.34 | 6.78 | 12.12 | 14.254 |
| 200 mg/L-1 | 21.79 | 22.58 | 18.03 | 19.21 | 20.00 | 20.322 |
| 200 mg/L-2 | 16.79 | 19.59 | 19.21 | 16.45 | 20.12 | 18.432 |
| 200 mg/L-3 | 16.47 | 15.49 | 14.78 | 18.21 | 19.20 | 16.83 |

1. Chlorophyll content a and b

| Treatments | Weight/g | Absorbance | | Chl a  (mg/g·FW) | Chl b  (mg/g·FW) |
| --- | --- | --- | --- | --- | --- |
|  |  | 665 nm | 649 nm |  |  |
| 0 mg/L-1 | 0.1475 | 0.983 | 0.501 | 0.707892 | 0.496163 |
| 0 mg/L-2 |  | 1.006 | 0.580 | 0.733243 | 0.429993 |
| 0 mg/L-3 |  | 1.019 | 0.546 | 0.717379 | 0.369831 |
| 12.5 mg/L-1 | 0.1414 | 1.108 | 0.562 | 0.814311 | 0.481867 |
| 12.5 mg/L-2 |  | 1.086 | 0.584 | 0.806714 | 0.489279 |
| 12.5 mg/L-3 |  | 1.079 | 0.586 | 0.844588 | 0.429901 |
| 25 mg/L-1 | 0.1635 | 1.275 | 0.636 | 0.696492 | 0.451450 |
| 25 mg/L-2 |  | 1.086 | 0.606 | 0.697672 | 0.423144 |
| 25 mg/L-3 |  | 1.079 | 0.586 | 0.844290 | 0.410936 |
| 50 mg/L-1 | 0.1427 | 1.048 | 0.527 | 0.755159 | 0.528353 |
| 50 mg/L-2 |  | 1.038 | 0.598 | 0.774491 | 0.460603 |
| 50 mg/L-3 |  | 1.043 | 0.562 | 0.793418 | 0.394660 |
| 100 mg/L-1 | 0.1358 | 0.841 | 0.426 | 0.748038 | 0.473903 |
| 100 mg/L-2 |  | 0.966 | 0.534 | 0.774635 | 0.445832 |
| 100 mg/L-3 |  | 0.989 | 0.526 | 0.667742 | 0.338675 |
| 200 mg/L-1 | 0.1427 | 0.798 | 0.403 | 0.573553 | 0.347134 |
| 200 mg/L-2 |  | 0.774 | 0.420 | 0.637680 | 0.327400 |
| 200 mg/L-3 |  | 0.845 | 0.430 | 0.603456 | 0.303616 |

1. Root length

| Treatments | Root length (cm) | | | | | |
| --- | --- | --- | --- | --- | --- | --- |
|  | 1 | 2 | 3 | 4 | 5 | Average |
| 0 mg/L-1 | 4.6 | 8.72 | 16.2 | 4.31 | 8.5 | 8.466 |
| 0 mg/L-2 | 2.9 | 9.7 | 9.52 | 9.3 | 2.56 | 6.796 |
| 0 mg/L-3 | 8.2 | 4.56 | 3.5 | 8.2 | 6.8 | 6.252 |
| 12.5 mg/L-1 | 16.1 | 7.1 | 5.6 | 9.2 | 4.5 | 8.5 |
| 12.5 mg/L-2 | 7.2 | 6.9 | 6.2 | 6.8 | 6.5 | 6.72 |
| 12.5 mg/L-3 | 10.4 | 5.9 | 9.81 | 5 | 5.2 | 7.262 |
| 25 mg/L-1 | 6.9 | 8.6 | 10.02 | 11.5 | 4.3 | 8.264 |
| 25 mg/L-2 | 11.1 | 8.5 | 4.6 | 6.6 | 3 | 6.76 |
| 25 mg/L-3 | 6.89 | 4.3 | 6.4 | 10.2 | 7.8 | 7.118 |
| 50 mg/L-1 | 8 | 4.3 | 11.5 | 6.5 | 7.2 | 7.5 |
| 50 mg/L-2 | 6.5 | 8.2 | 7.8 | 4.8 | 12.8 | 8.02 |
| 50 mg/L-3 | 9.1 | 6.1 | 13.5 | 12.2 | 6.2 | 9.42 |
| 100 mg/L-1 | 10.01 | 7.8 | 13.21 | 14.15 | 10.2 | 11.074 |
| 100 mg/L-2 | 9.1 | 10.15 | 20.2 | 6 | 3.2 | 9.73 |
| 100 mg/L-3 | 5.3 | 9.21 | 6.2 | 12.15 | 12.7 | 9.112 |
| 200 mg/L-1 | 7.85 | 14.5 | 11.72 | 19.2 | 9.99 | 12.652 |
| 200 mg/L-2 | 12.12 | 7.95 | 8 | 10.12 | 8.98 | 9.434 |
| 200 mg/L-3 | 11.12 | 12.15 | 9.15 | 10 | 12 | 10.884 |

1. Root activity

| Treatments | Weight/g | Absorbance  (485 nm) | Tetrazolium reduction  (mg) | Reduction strength of tetrazolium (mg·g^-1^·h^-1)^ |
| --- | --- | --- | --- | --- |
| 0 mg/L-1 | 0.2222 | 0.493 | 0.671622 | 1.060561 |
| 0 mg/L-2 |  | 0.493 | 0.671622 | 1.060561 |
| 0 mg/L-3 |  | 0.494 | 0.672973 | 1.062695 |
| 12.5 mg/L-1 | 0.2055 | 0.519 | 0.706757 | 1.206739 |
| 12.5 mg/L-2 |  | 0.519 | 0.706757 | 1.206739 |
| 12.5 mg/L-3 |  | 0.520 | 0.708108 | 1.209046 |
| 25 mg/L-1 | 0.2108 | 0.617 | 0.839189 | 1.396833 |
| 25 mg/L-2 |  | 0.618 | 0.840541 | 1.399082 |
| 25 mg/L-3 |  | 0.617 | 0.839189 | 1.396833 |
| 50 mg/L-1 | 0.2105 | 0.513 | 0.698649 | 1.16456 |
| 50 mg/L-2 |  | 0.514 | 0.7 | 1.166813 |
| 50 mg/L-3 |  | 0.512 | 0.697297 | 1.162307 |
| 100 mg/L-1 | 0.1989 | 0.651 | 0.885135 | 1.561457 |
| 100 mg/L-2 |  | 0.653 | 0.887838 | 1.566224 |
| 100 mg/L-3 |  | 0.649 | 0.882432 | 1.556689 |
| 200 mg/L-1 | 0.2012 | 0.588 | 0.8 | 1.395138 |
| 200 mg/L-2 |  | 0.589 | 0.801351 | 1.397495 |
| 200 mg/L-3 |  | 0.587 | 0.798649 | 1.392781 |

1. SOD activity

| Treatments | Weight/g  Leaves/Roots | Absorbance  (560 nm) | Leaves’ SOD activity (U/g·FW) | Absorbance  (560 nm) | Roots’ SOD activity (U/g·FW) |
| --- | --- | --- | --- | --- | --- |
| 0 mg/L-1 | 0.1994/0.1998 | 0.580 | 67.27008 | 0.379 | 139.6976 |
| 0 mg/L-2 |  | 0.600 | 59.48233 | 0.489 | 93.08472 |
| 0 mg/L-3 |  | 0.590 | 64.34195 | 0.379 | 139.6976 |
| 12.5 mg/L-1 | 0.1984/0.2013 | 0.450 | 121.1837 | 0.394 | 132.3477 |
| 12.5 mg/L-2 |  | 0.480 | 109.9829 | 0.391 | 133.6095 |
| 12.5 mg/L-3 |  | 0.450 | 119.2073 | 0.394 | 132.3477 |
| 25 mg/L-1 | 0.1948/0.1999 | 0.510 | 99.0959 | 0.450 | 109.5563 |
| 25 mg/L-2 |  | 0.480 | 111.0534 | 0.398 | 131.5805 |
| 25 mg/L-3 |  | 0.530 | 89.51089 | 0.450 | 109.5563 |
| 50 mg/L-1 | 0.1926/0.2011 | 0.510 | 98.83772 | 0.451 | 108.4815 |
| 50 mg/L-2 |  | 0.500 | 103.0081 | 0.442 | 112.2707 |
| 50 mg/L-3 |  | 0.540 | 84.83155 | 0.451 | 108.4815 |
| 100 mg/L-1 | 0.1983/0.2006 | 0.560 | 74.79911 | 0.496 | 89.75904 |
| 100 mg/L-2 |  | 0.460 | 118.2744 | 0.378 | 139.5626 |
| 100 mg/L-3 |  | 0.590 | 63.6183 | 0.496 | 89.75904 |
| 200 mg/L-1 | 0.1936/0.2001 | 0.520 | 96.25278 | 0.454 | 107.7543 |
| 200 mg/L-2 |  | 0.490 | 108.4227 | 0.444 | 111.9855 |
| 200 mg/L-3 |  | 0.490 | 108.0512 | 0.454 | 107.7543 |

1. POD activity

| Treatments | Weight/g  Leaves/Roots | Absorbance  (470 nm) | Leaves’ POD activity (U/g·min) | Absorbance  (470 nm) | Roots’ POD activity (U/g·min) |
| --- | --- | --- | --- | --- | --- |
| 0 mg/L-1 | 0.1998/0.1946 | 0.256 | 7687.688 | 0.256 | 7893.114 |
| 0 mg/L-2 |  | 0.199 | 5975.976 | 0.455 | 7014.388 |
| 0 mg/L-3 |  | 0.199 | 5975.976 | 0.698 | 7173.69 |
| 12.5 mg/L-1 | 0.2013/0.1930 | 0.299 | 8912.072 | 0.299 | 9295.337 |
| 12.5 mg/L-2 |  | 0.286 | 8524.59 | 0.585 | 9093.264 |
| 12.5 mg/L-3 |  | 0.286 | 8524.59 | 0.807 | 8362.694 |
| 25 mg/L-1 | 0.1999/0.1906 | 0.438 | 13146.57 | 0.438 | 13788.04 |
| 25 mg/L-2 |  | 0.360 | 10805.4 | 0.798 | 12560.34 |
| 25 mg/L-3 |  | 0.360 | 10805.4 | 1.158 | 12151.1 |
| 50 mg/L-1 | 0.2011/0.1926 | 0.405 | 12083.54 | 0.405 | 12616.82 |
| 50 mg/L-2 |  | 0.354 | 10561.91 | 0.759 | 11822.43 |
| 50 mg/L-3 |  | 0.354 | 10561.91 | 1.113 | 11557.63 |
| 100 mg/L-1 | 0.2006/0.1956 | 0.290 | 8673.978 | 0.290 | 8895.706 |
| 100 mg/L-2 |  | 0.243 | 7268.195 | 0.533 | 8174.847 |
| 100 mg/L-3 |  | 0.243 | 7268.195 | 0.776 | 7934.56 |
| 200 mg/L-1 | 0.2001/0.1961 | 0.343 | 10284.86 | 0.343 | 10494.65 |
| 200 mg/L-2 |  | 0.235 | 7046.477 | 0.578 | 8842.427 |
| 200 mg/L-3 |  | 0.235 | 7046.477 | 0.813 | 8291.688 |

1. CAT activity

| Treatments | Weight/g  Leaves/Roots | Absorbance  (240 nm) | Leaves’ CAT activity (U/g·min) | Absorbance  (240 nm) | Roots’ CAT activity (U/g·min) |
| --- | --- | --- | --- | --- | --- |
| 0 mg/L-1 | 0.1994/0.1998 | 0.056 | 4.212638 | 0.063 | 4.72973 |
| 0 mg/L-2 |  | 0.061 | 4.563691 | 0.075 | 5.655656 |
| 0 mg/L-3 |  | 0.056 | 4.212638 | 0.063 | 4.72973 |
| 12.5 mg/L-1 | 0.1984/0.2013 | 0.051 | 3.855847 | 0.058 | 4.321908 |
| 12.5 mg/L-2 |  | 0.057 | 4.334677 | 0.071 | 5.290611 |
| 12.5 mg/L-3 |  | 0.051 | 3.855847 | 0.058 | 4.321908 |
| 25 mg/L-1 | 0.1906/0.1999 | 0.059 | 4.643232 | 0.067 | 5.027514 |
| 25 mg/L-2 |  | 0.061 | 4.826863 | 0.076 | 5.677839 |
| 25 mg/L-3 |  | 0.059 | 4.643232 | 0.067 | 5.027514 |
| 50 mg/L-1 | 0.1926/0.2011 | 0.050 | 3.894081 | 0.058 | 4.326206 |
| 50 mg/L-2 |  | 0.055 | 4.283489 | 0.068 | 5.096967 |
| 50 mg/L-3 |  | 0.050 | 3.894081 | 0.058 | 4.326206 |
| 100 mg/L-1 | 0.1956/0.2006 | 0.049 | 3.757669 | 0.059 | 4.411765 |
| 100 mg/L-2 |  | 0.048 | 3.641529 | 0.064 | 4.810568 |
| 100 mg/L-3 |  | 0.049 | 3.757669 | 0.059 | 4.411765 |
| 200 mg/L-1 | 0.1961/0.2001 | 0.059 | 4.513004 | 0.081 | 6.071964 |
| 200 mg/L-2 |  | 0.055 | 4.232534 | 0.076 | 5.722139 |
| 200 mg/L-3 |  | 0.059 | 4.513004 | 0.081 | 6.071964 |

1. Proline content

| Treatments | Weight/g  Leaves/Roots | Absorbance  (520 nm) | Leaves’ proline content (μg/g) | Absorbance  (520 nm) | Roots’ proline content (μg/g) |
| --- | --- | --- | --- | --- | --- |
| 0 mg/L-1 | 0.2548/0.1998 | 0.273 | 19.38931 | 0.140 | 13.70576 |
| 0 mg/L-2 |  | 0.290 | 20.49393 | 0.137 | 13.45717 |
| 0 mg/L-3 |  | 0.270 | 19.19438 | 0.164 | 15.6945 |
| 12.5 mg/L-1 | 0.2598/0.2013 | 0.293 | 20.2907 | 0.150 | 14.4261 |
| 12.5 mg/L-2 |  | 0.292 | 20.22697 | 0.158 | 15.08407 |
| 12.5 mg/L-3 |  | 0.28 | 19.46225 | 0.189 | 17.63373 |
| 25 mg/L-1 | 0.2439/0.1999 | 0.293 | 21.61346 | 0.155 | 14.94125 |
| 25 mg/L-2 |  | 0.303 | 22.29228 | 0.169 | 16.10077 |
| 25 mg/L-3 |  | 0.285 | 21.07041 | 0.203 | 18.91674 |
| 50 mg/L-1 | 0.2597/0.2011 | 0.281 | 19.53349 | 0.138 | 13.4525 |
| 50 mg/L-2 |  | 0.299 | 20.68102 | 0.132 | 12.95853 |
| 50 mg/L-3 |  | 0.268 | 18.70472 | 0.159 | 15.1814 |
| 100 mg/L-1 | 0.2578/0.2006 | 0.263 | 18.52147 | 0.130 | 12.82576 |
| 100 mg/L-2 |  | 0.267 | 18.77835 | 0.114 | 11.50522 |
| 100 mg/L-3 |  | 0.260 | 18.3288 | 0.137 | 13.4035 |
| 200 mg/L-1 | 0.2561/0.2001 | 0.215 | 15.54132 | 0.120 | 12.03041 |
| 200 mg/L-2 |  | 0.210 | 15.21808 | 0.097 | 10.12739 |
| 200 mg/L-3 |  | 0.245 | 17.48075 | 0.116 | 11.69945 |

1. MDA content

| Treatments | Weight/g  Leaves/Roots | Absorbance  (532 nm- 600 nm) | Leaves’ MDA content (nmol/g·FW) | Absorbance  (532 nm- 600 nm) | Roots’ MDA content (nmol/g·FW) |
| --- | --- | --- | --- | --- | --- |
| 0 mg/L-1 | 0.1994/0.1998 | 0.047 | 0.076039 | 0.068 | 0.109794 |
| 0 mg/L-2 |  | 0.049 | 0.079275 | 0.059 | 0.095262 |
| 0 mg/L-3 |  | 0.045 | 0.072803 | 0.074 | 0.119481 |
| 12.5 mg/L-1 | 0.1984/0.2013 | 0.032 | 0.052032 | 0.056 | 0.089745 |
| 12.5 mg/L-2 |  | 0.038 | 0.061788 | 0.048 | 0.076924 |
| 12.5 mg/L-3 |  | 0.036 | 0.058536 | 0.070 | 0.112181 |
| 25 mg/L-1 | 0.1948/0.1999 | 0.047 | 0.077835 | 0.063 | 0.10167 |
| 25 mg/L-2 |  | 0.049 | 0.081147 | 0.059 | 0.095215 |
| 25 mg/L-3 |  | 0.050 | 0.082803 | 0.076 | 0.122649 |
| 50 mg/L-1 | 0.1926/0.2011 | 0.051 | 0.085424 | 0.065 | 0.104272 |
| 50 mg/L-2 |  | 0.053 | 0.088774 | 0.063 | 0.101063 |
| 50 mg/L-3 |  | 0.049 | 0.082074 | 0.075 | 0.120313 |
| 100 mg/L-1 | 0.1983/0.2006 | 0.054 | 0.087849 | 0.067 | 0.107748 |
| 100 mg/L-2 |  | 0.065 | 0.105744 | 0.078 | 0.125438 |
| 100 mg/L-3 |  | 0.064 | 0.104117 | 0.081 | 0.130262 |
| 200 mg/L-1 | 0.1936/0.2001 | 0.063 | 0.104978 | 0.076 | 0.122527 |
| 200 mg/L-2 |  | 0.058 | 0.096647 | 0.070 | 0.112854 |
| 200 mg/L-3 |  | 0.054 | 0.089981 | 0.078 | 0.125751 |

1. Leaves (Evan’s Blue assay)

| Treatments | 1. Absorbance   (600 nm) | 1. Absorbance   (600 nm) | 1. Absorbance   (600 nm) |
| --- | --- | --- | --- |
| 0 mg/L-1 | 0.094 | 0.129 | 0.106 |
| 12.5 mg/L-1 | 0.085 | 0.122 | 0.087 |
| 25 mg/L-1 | 0.112 | 0.133 | 0.098 |
| 50 mg/L-1 | 0.103 | 0.132 | 0.117 |
| 100 mg/L-1 | 0.157 | 0.139 | 0.161 |
| 200 mg/L-1 | 0.162 | 0.141 | 0.192 |

1. Water absorption

| Treatments | Initial mass/g | 2h/g | 4h/g | 6h/g | 8h/g | 10h/g | 24h/g | 36h/g |
| --- | --- | --- | --- | --- | --- | --- | --- | --- |
| 0 mg/L-1 | 30.8 | 54.9 | 56.8 | 61.4 | 62.5 | 63.6 | 83.2 | 107.8 |
| 0 mg/L-2 | 34.3 | 58.6 | 60.1 | 66.7 | 67.2 | 68.9 | 87.9 | 113.2 |
| 0 mg/L-3 | 32.7 | 56.1 | 59.3 | 64.3 | 65.2 | 66.6 | 86.2 | 111.5 |
| 12.5 mg/L-1 | 33.3 | 72.3 | 74.5 | 81.2 | 83.5 | 84 | 107 | 131.8 |
| 12.5 mg/L-2 | 32.8 | 67 | 73.6 | 80 | 83 | 85 | 106.4 | 132.1 |
| 12.5 mg/L-3 | 31.5 | 69.3 | 72.2 | 79.1 | 83.1 | 84.2 | 106.9 | 131.4 |
| 25 mg/L-1 | 30 | 67.6 | 70.8 | 74.5 | 77.2 | 77.7 | 103.9 | 140.9 |
| 25 mg/L-2 | 29.4 | 65 | 68.5 | 75.1 | 79 | 80.1 | 106.1 | 143 |
| 25 mg/L-3 | 30.4 | 68.2 | 71.1 | 73.2 | 78.2 | 79.2 | 105.2 | 142.1 |
| 50 mg/L-1 | 31.6 | 70.9 | 71.6 | 72.4 | 75.2 | 75.6 | 97.9 | 120.4 |
| 50 mg/L-2 | 29 | 68.4 | 69.4 | 70.1 | 74.1 | 75 | 98.2 | 121.5 |
| 50 mg/L-3 | 33.7 | 72.4 | 73.5 | 74.5 | 78.3 | 79.2 | 102.6 | 125.6 |
| 100 mg/L-1 | 35.3 | 78.1 | 83.7 | 86.4 | 89.1 | 91 | 115.6 | 148.2 |
| 100 mg/L-2 | 34.4 | 77.6 | 82.5 | 86.2 | 88.7 | 90.7 | 114.2 | 147.5 |
| 100 mg/L-3 | 29.6 | 71.2 | 76.1 | 80.1 | 89.4 | 90.9 | 115 | 148.1 |
| 200 mg/L-1 | 28.5 | 64.3 | 78.3 | 79.1 | 84.2 | 85.2 | 97.6 | 133.6 |
| 200 mg/L-2 | 32 | 68.5 | 82.5 | 83.2 | 88.3 | 89.1 | 101.2 | 137.8 |
| 200 mg/L-3 | 31.4 | 69 | 81.1 | 82.1 | 87 | 88.4 | 100.9 | 136.9 |
